# Transcriptome analysis reveals the effects of sugar metabolism and auxin and cytokinin signaling pathways on root growth and development of grafted apple

**DOI:** 10.1186/s12864-016-2484-x

**Published:** 2016-02-29

**Authors:** Guofang Li, Juanjuan Ma, Ming Tan, Jiangping Mao, Na An, Guangli Sha, Dong Zhang, Caiping Zhao, Mingyu Han

**Affiliations:** College of Horticulture, Northwest A & F University, Yangling, Shaanxi 712100 China; Institute of agricultural science, Qingdao, Shandong 266000 China

**Keywords:** Grafted apple, Root growth and development, Sugar metabolism, Auxin signaling, CKs signaling, Cell cycle

## Abstract

**Background:**

The root architecture of grafted apple (*Malus* spp.) is affected by various characteristics of the scions. To provide information on the molecular mechanisms underlying this influence, we examined root transcriptomes of *M. robusta* rootstock grafted with scions of wild-type (WT) apple (*M. spectabilis*) and a more-branching (MB) mutant at the branching stage.

**Results:**

The growth rate of rootstock grafted MB was repressed significantly, especially the primary root length and diameter, and root weight. Biological function categories of differentially expressed genes were significantly enriched in processes associated with hormone signal transduction and intracellular activity, with processes related to the cell cycle especially down-regulated. Roots of rootstock grafted with MB scions displayed elevated auxin and cytokinin contents and reduced expression of *MrPIN1*, *MrARF*, *MrAHP*, most *MrCRE1* genes, and cell growth-related genes *MrGH3*, *MrSAUR* and *MrTCH4*. Although auxin accumulation and transcription of *MrPIN3*, *MrALF1* and *MrALF4* tended to induce lateral root formation in MB-grafted rootstock, the number of lateral roots was not significantly changed. Sucrose, fructose and glucose contents were not decreased in MB-grafted roots compared with those bearing WT scions, but glycolysis and tricarboxylic acid cycle metabolic activities were repressed. Root resistance and nitrogen metabolism were reduced in MB-grafted roots as well.

**Conclusions:**

Our findings suggest that root growth and development of rootstock are mainly influenced by sugar metabolism and auxin and cytokinin signaling pathways. This study provides a basis that the characteristics of scions are related to root growth and development, resistance and activity of rootstocks.

**Electronic supplementary material:**

The online version of this article (doi:10.1186/s12864-016-2484-x) contains supplementary material, which is available to authorized users.

## Background

In terms of nutritional value and economic importance, apple (*Malus domestica* Borkh.), grape, orange and banana are globally the most predominant fruit crops. Among these four fruit crops, apple is king. Apple production relies heavily on grafting, a technique that combines well-adapted rootstocks with high-quality scions. Various aspects of scion growth and development, such as plant height, fruiting rate, resistance, physiological and biochemical characteristics and environmental adaptability [1–4], are invariably influenced by the rootstock. Grafting experiments have revealed that plant vascular systems function as transportation corridors for hormones, sugars and RNA molecules [5–7]. Plant vascular systems including xylem and phloem tissues play crucial roles in the transportation of water, minerals and organics substances, and serve as the junction between aboveground and belowground tissues [5, 8]. Extensive research has demonstrated that grafted gain-of-function transcripts can impact tissue development, thereby influencing features such as leaf shape and root architecture [1, 2, 9–11].

The developmental plasticity of roots, which are mainly composed of lateral roots (LRs) and primary roots (PRs), is regulated by hormonal signals and nutrients [12–15]. The relationship of cytokinins (CKs), brassinolide (BR), abscisic acid (ABA), gibberellins (GAs), ethylene and strigolactones are related to auxin biosynthesis, transport, distribution and/or signalling is relatively clear [16–19]. As nutrient components, the main targets of sugar signals are auxin, ABA and CK signalling processes [20, 21].

Auxin transport and signalling play essential roles in PR growth and LR formation [22–24]. CKs, which are antagonistic to auxin, repress PR growth and LR initiation by suppressing cell differentiation [25–27]. This antagonism between auxin and CKs in PRs was mediated by SHORT HYPOCOTYL 2 (SHY2) [28, 29]. Arabidopsis response regulator 1 (ARR1), which activates CK signaling, binds directly to the *SHY2* promoter region. In response to auxin, SHY2 degradation is induced to enable auxin transport and distribution. During LR formation, signaling components including histidine kinases (AHKs) and ARRs are also involved in the inhibitory effect of CK [30, 31], which research has shown is unaffected by the addition of auxin [32].

In roots, cell cycle and differentiation-related genes, such as CYCA2;1, CYCA2;4, CYCB1;1, CYCD1;1, CYCD3;2 and CDKB2;1, are activated by auxin [33–35]. Several root development-related genes identified from studies of root phenotypic mutants, are responsible for meristematic activity [36]. For instance, *SHORT ROOT* (*SHR*) is specifically expressed in root column vascular tissues to regulate root longitudinal growth [37, 38]. Even when treated with exogenous auxin, the *aberrant lateral root formation-4* (*alf4*) only has normal PRs but no LRs [39].

In grafted apple seedlings, rootstock root architecture, resistance and root regeneration are affected by the scion [40, 41]. The molecular mechanisms underlying these effects are poorly understood. With the availability of the apple complete genome sequence and the advance of next-generation high-throughput RNA sequencing (RNA-seq) technology, genome-wide transcriptome analysis can be applied to study gene expression patterns in different tissues under various conditions during apple root growth and development.

After grafting scions with consistent genetic backgrounds onto identical rootstock materials, we used RNA-seq of roots to analyze gene expression patterns of development-related biological processes. A combined analysis of plant growth dynamics and hormone and sugar contents in roots indicated that scion characteristics can influence rootstock phenotypes, which were mainly regulated by auxin and CK signaling pathways. Root activity and resistance were also influenced. These results may be helpful for further understanding of the mechanisms that cause grafting and internal conditions to influence root growth and development.

## Results

### Phenotypic changes in grafted seedlings

**Fig. 1:**
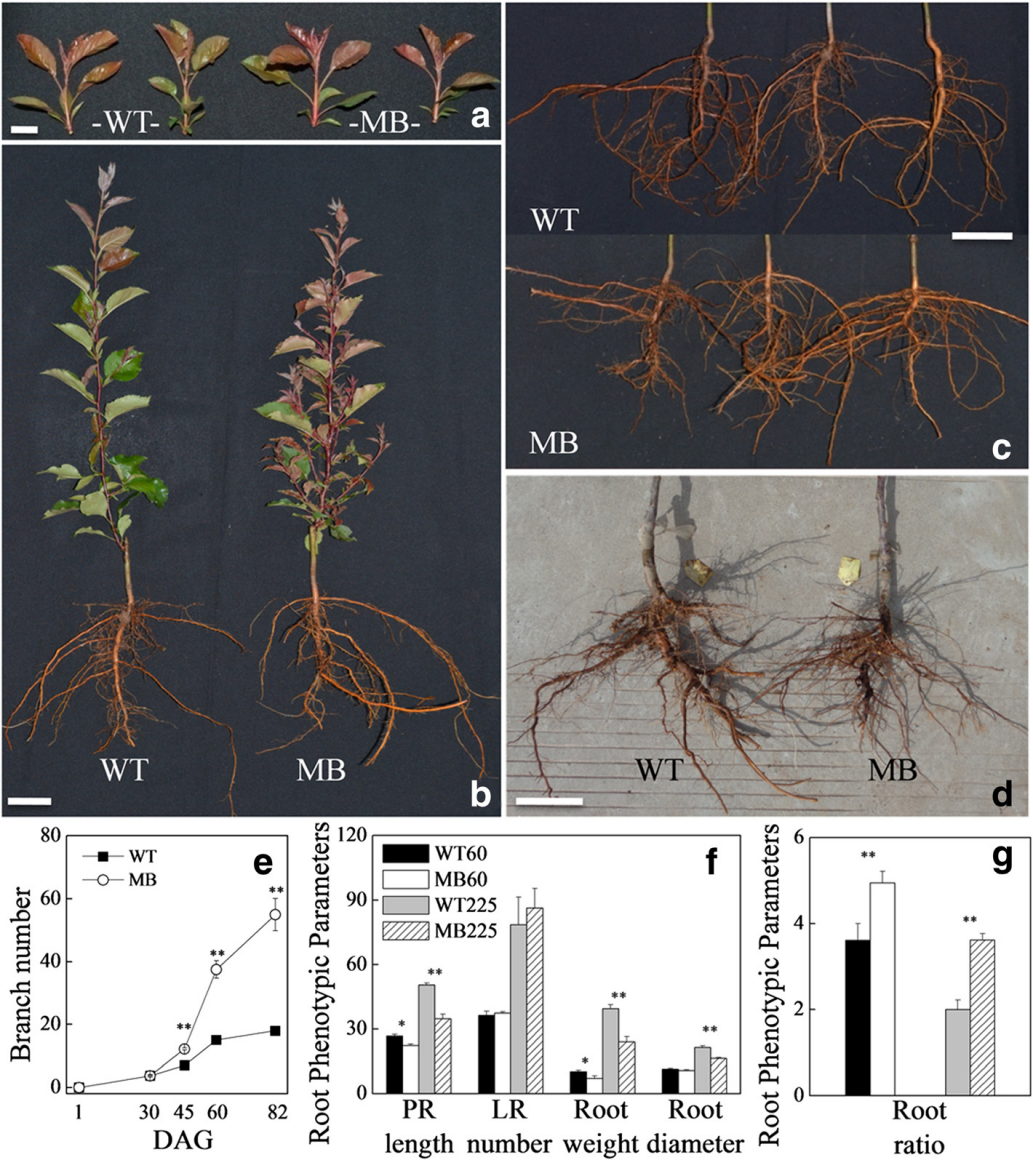
Phenotypic changes in wild-type (WT) and more-branching (MB) grafted apple (*M. spectabilis*) scions and *M. robusta* rootstock. **a**–**b** Branching phenotypes of WT and MB grafted scions at different growth stages (**a**, 25 days after scion bud germination [DAG]; **b**, 60 DAG); **c**–**d** Root phenotypes at the branching stage (60 and 225 DAG). **e**–**g** Comparison of branching number, root phenotypic parameters and root ratio. Data are means ± SE (*n* = 10). Root ratio was assessed by the relative proportion of LR number to total root weight. Significant differences (*P < 0.05 and **P < 0.01) are based on Student’s *t*-test. Scale bars = 2.0 cm (**a**), 5.0 cm (**b**), 10.0 cm (**c**) and 10.0 cm (**d**)

To quantify the effect of scions on rootstock phenotype, we grafted seedlings of wild type (WT) *M. spectabilis* ‘Bly114’ and its more-branching mutant (MB) onto identical *M. robusta* rootstocks. Scions of young plants displayed similar phenotypes, with obvious differences appearing at the branching stage (60 days after scion bud germination) (Fig. 1b and e). PR length and diameter and root fresh weight were reduced in MB-bearing rootstock compared with WT-grafted material, whereas the number of LRs was slightly but not significantly higher (Fig. 1c, d and f). To assess the relative proportion of LR growth to total root growth, we used the ratio of LR number to total root weight. As shown in Fig. 1g, this ratio was higher in MB-bearing rootstock. This ratio progressively decreased in both MB- and WT-bearing rootstocks as the growth period was extended. These results indicate that root growth, particularly the primary root, is the main process.

### Quantitative analysis of sugars, photosynthetic parameters and hormones

**Fig. 2:**
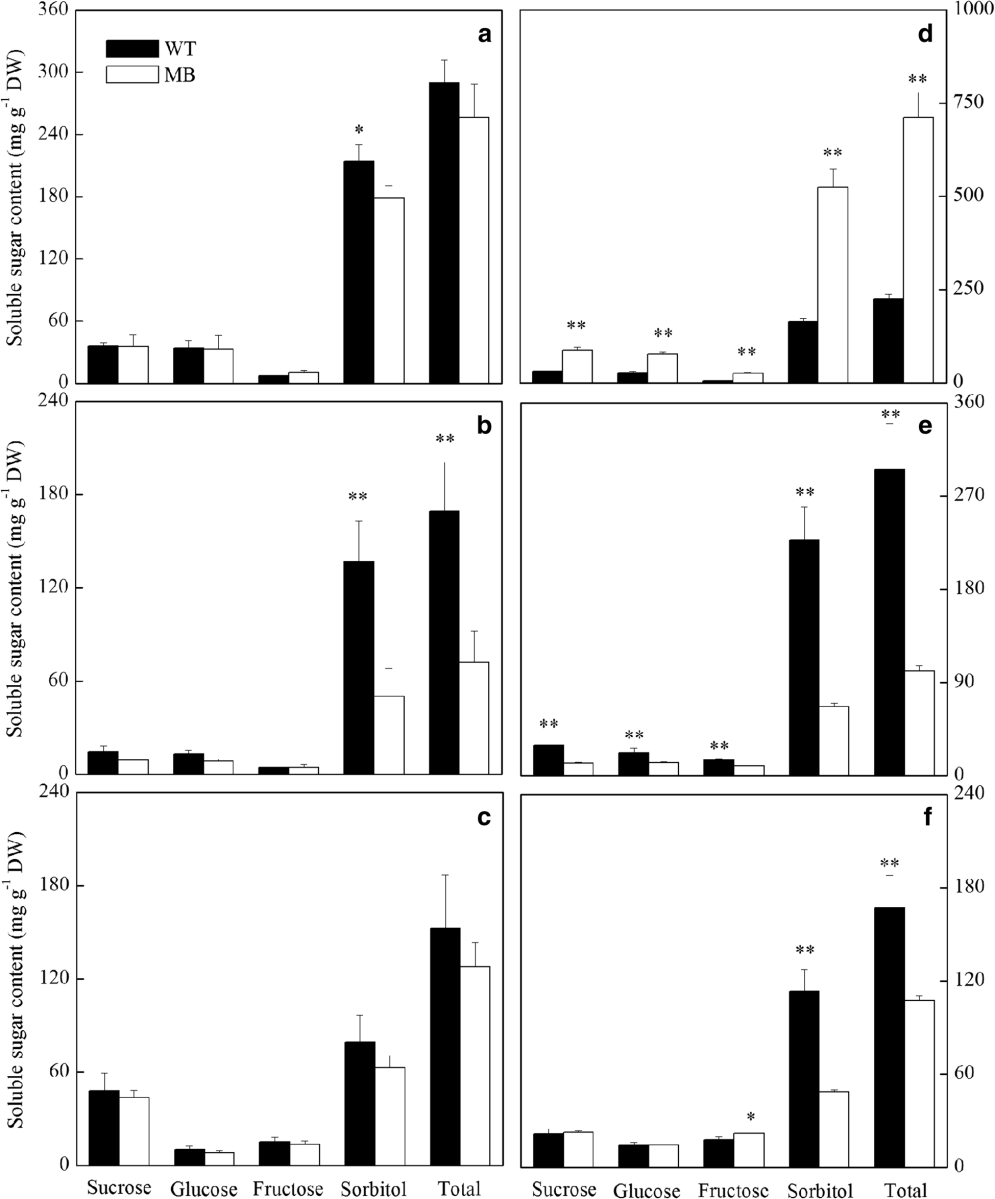
Quantification of soluble sugars in WT and MB grafted apples. Sugar content in leaves (**a**, **d**), stems (**b**, **e**) and roots (**c**, **f**) at the early growth stage (25 DAG, **a**–**c**) and the branching stage (60 DAG, **d**–**f**). Values are means ± SE (*n* = 3). Significant differences (*P < 0.05 and **P < 0.01) are based on Student’s *t*-tests

To evaluate nutrient levels, we measured levels of soluble sugars, including sucrose, glucose, fructose and sorbitol, in roots, stems and leaves at early growth and branching stages (25 and 60 days after scion bud germination, respectively) (Fig. 2). At the early growth stage (Fig. 2a-c), the only significant difference was that sorbitol levels were lower in stems of MB than in WT scions (Fig. 2b). At the branching stage, levels of all soluble sugars in the two scions were highest in MB leaves and lowest in MB stems (Fig. 2d and e), which indicates that sugars were concentrated in MB leaves with lower output. Sorbitol content was relatively lower in roots of MB-bearing rootstock while fructose content was slightly higher (Fig. 2f). Compared with WT scions, total soluble sugar content of stems and roots was lower in MB scions and that of leaves was higher.

Moreover, net photosynthetic efficiency (*Pn*) was higher in WT than MB leaves and was not balanced by the lower stomatal conductance (*Gs*) needed for equal intercellular CO_2_ concentration (*Ci*) (Additional file 1).

**Fig. 3:**
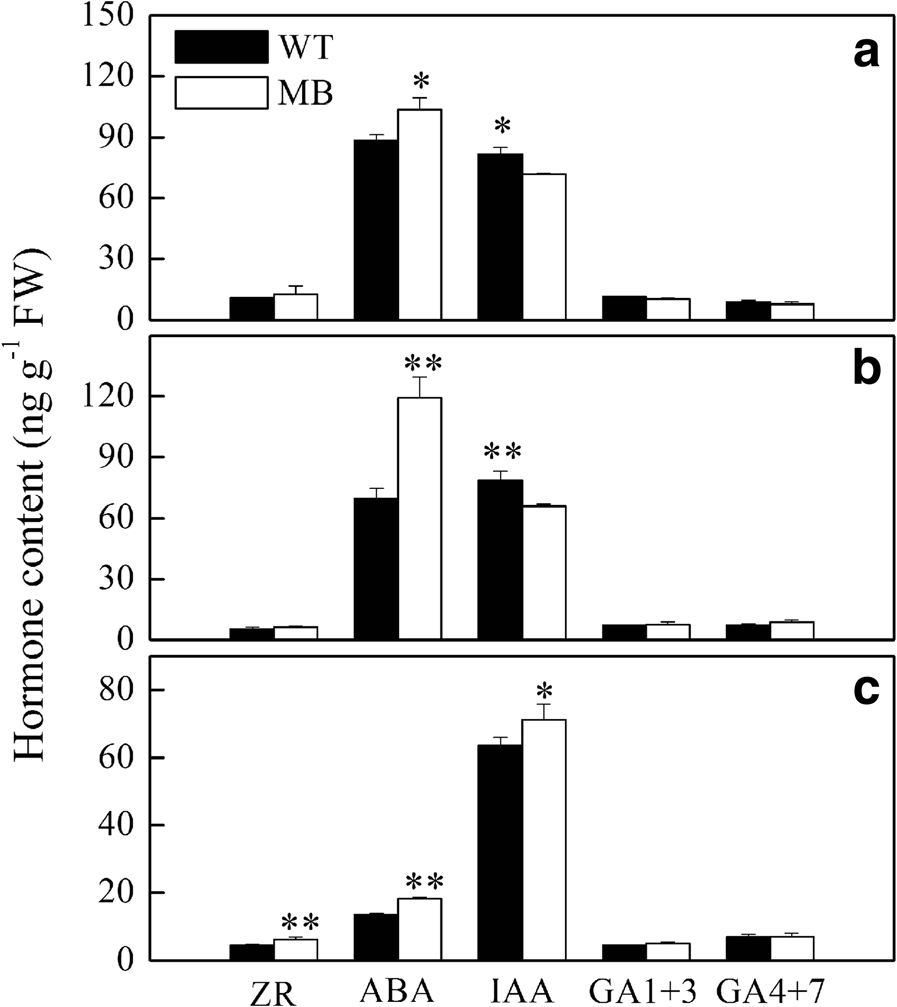
Quantification of free ZR, IAA, GAs and ABA in WT and MB grafted apple. Hormone content in shoot tips (**a**), stems (**b**) and roots (**c**) at the branching stage (60 DAG). Values are means ± SE (*n* = 3, with three technological duplicates). Significant differences (*P < 0.05 and **P < 0.01) are based on Student’s *t*-tests

Zeatin riboside (ZR), ABA and indole acetic acid (IAA) contents were significantly higher in roots of MB-bearing rootstock, while GAs contents were unchanged (Fig. 3c). The ABA content of shoot tips, stems and roots of grafted MB was obviously higher than that of grafted WT, whereas IAA content was higher in stems and shoot tips, but not roots, of grafted WT (Fig. 3a-c). These data demonstrate that higher IAA and ZR in roots of MB-bearing rootstock should be closely related to its root anatomy (Fig. 1d).

### Tissue sampling, RNA-seq and analysis of differentially expressed genes

To gain global insights into the molecular mechanisms responsible for the different phenotypes (Fig. 1c-e), root apex samples without LRs [42] were collected from WT and MB for transcriptomic analysis at the branching stage. To ensure data reliability, two samples of each type of seedling were sequenced.

**Fig. 4:**
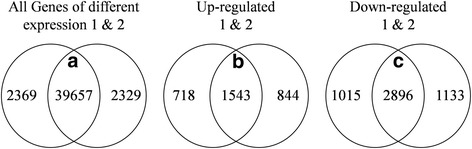
Venn diagrams of all genes of different expression and DEGs between roots of grafted WT and MB apple. **a** The intersection of Venn diagram indicating that all genes of different expression were identified both in two difference analysis pairs (WT-1-VS-MB-1 and WT-2-VS-MB-2). **b**-**c** The intersection of Venn diagram indicating that genes were up- and down-regulated both in two difference analysis pairs

A statistical summary of RNA-seq results is given in Table 1. The genomic mapping rate was greater than 77.49 %, with the gene mapping rate reaching 61.32-61.66 %. Identification of differentially expressed genes (DEGs) was based on the criteria of false discovery rate (FDR) < 0.001, |log_2_Ratio| ≥ 1, and Reads Per kb per Million mapped reads (RPKM) ≥ 1 at least in one sample. Venn diagrams of all genes of different expression and DEGs in roots of WT- and MB-bearing rootstock are shown in Fig. 4. Most expressed genes had obviously similar expression levels between the two scion types, suggesting that MB had only a narrow influence on the rootstock. Among DEGs, 2896 were down-regulated and 1543 were up-regulated in root grafted MB.

**Table 1:** Summary of RNA sequencing data from roots of *M. robusta* rootstock grafted with wild-type (WT) and more-branching (MB) *M. spectabilis* scions

Sample name	Clean reads	Genome map rate	Gene map rate	Expressed gene
WT- 1	47335946	77.86 %	61.32 %	38653
WT- 2	47142182	78.24 %	61.58 %	38505
MB- 1	47137442	77.49 %	61.66 %	39377
MB- 2	47084672	77.70 %	61.65 %	39296

**Fig. 5:**
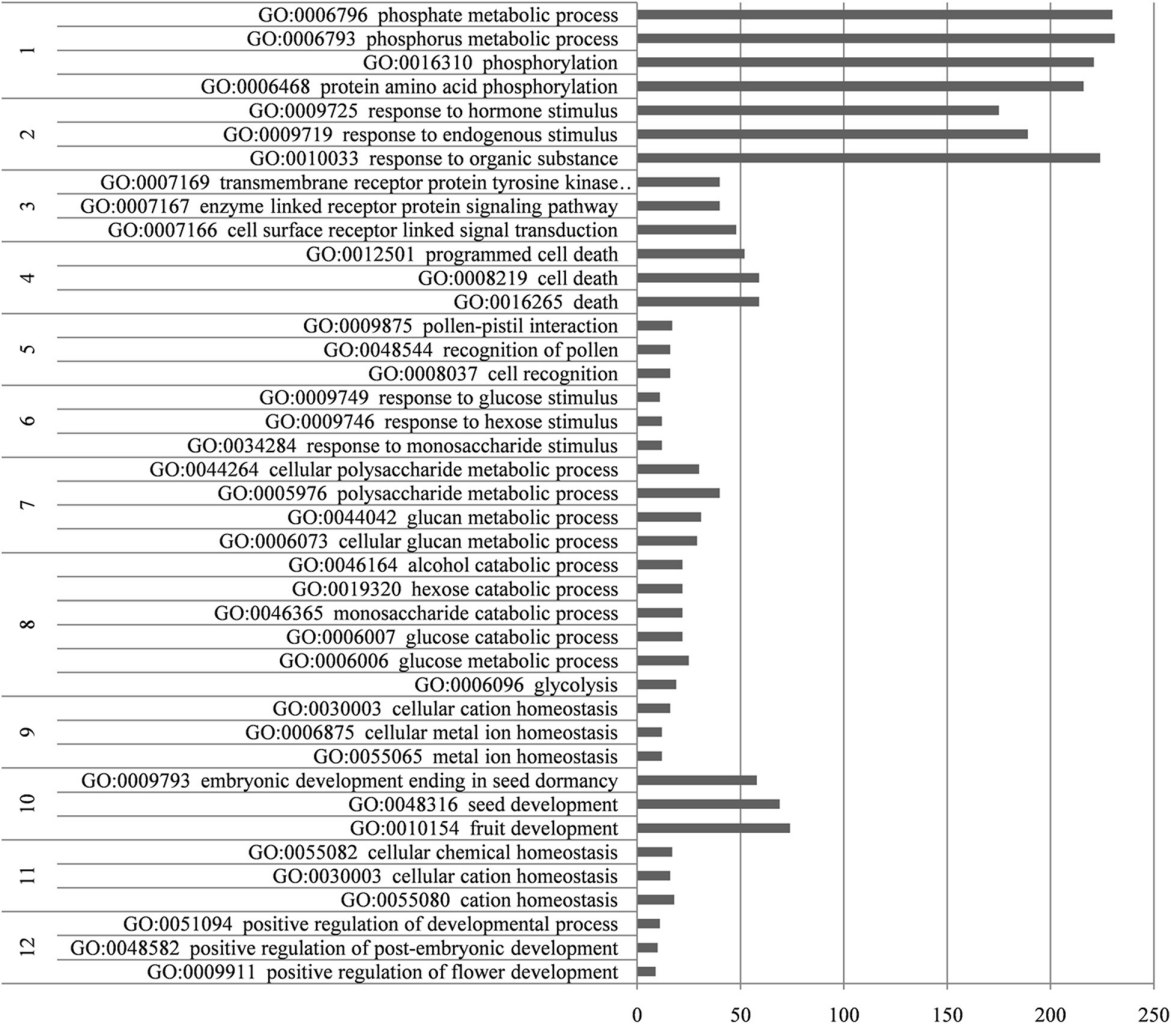
Clusters of annotated GO terms in the biological process category enriched in DEGs between roots of grafted WT and MB apple. DEGs were classified into specific biological process categories using DAVID with high classification stringency (*P* < 0.05). The horizontal ordinate represents the number of genes in the category

Gene Ontology (GO) and Kyoto Encyclopedia of Genes and Genomes (KEGG) pathway enrichment analyses were used to respectively identify biological processes and biological functions enriched in DEGs. Significantly enriched GO biological terms included those in the categories of sugar and phosphate metabolic processes (cluster 1, 7 and 8), response to stimulus and signaling (cluster 2, 3, 5 and 6), cellular activity (cluster 4, 9 and 11) and specific development processes (cluster 10 and 12) (Fig. 5). Furthermore, down-regulated genes were obviously enriched to signal transduction, metabolic process and cellular activity (Additional file 2). The most heavily enriched KEGG pathways were related to hormone signal transduction, specific metabolic processes and biosynthesis (Table 2). To evaluate cellular events, we compiled a list of DEGs with pathway annotations related to hormone signaling pathways and intracellular activity (Table 3). In this list, 61.70 % of genes, primarily falling into pathway categories of hormone signal transduction, RNA polymerase, brassinosteroid biosynthesis, aminoacyl-tRNA biosynthesis, histidine metabolism and ribosome biogenesis in eukaryotes, were down-regulated.

**Table 2:** KEGG pathway enrichment analysis of DEGs (*P* < 0.05)

Pathway	DEGs with pathway annotation (2774) (1612)	All genes with pathway annotation (35611) (35611)	Q-value	Pathway ID
Plant-pathogen interaction	653 (16.68 %)	3658 (10.27 %)	2.653051e-37	ko04626
Plant hormone signal transduction	312 (7.97 %)	2107 (5.92 %)	1.177574e-06	ko04075
Flavonoid biosynthesis	108 (2.76 %)	666 (1.87 %)	1.030723e-03	ko00941
Zeatin biosynthesis	54 (1.38 %)	310 (0.87 %)	1.385799e-02	ko00908
Flavone and flavonol biosynthesis	51 (1.3 %)	306 (0.86 %)	4.068123e-02	ko00944
Biosynthesis of secondary metabolites	621 (15.86 %)	5111 (14.35 %)	4.068123e-02	ko01110
ABC transporters	57 (1.46 %)	358 (1.01 %)	4.068123e-02	ko02010
Galactose metabolism	39 (1 %)	226 (0.63 %)	4.068123e-02	ko00052
Carotenoid biosynthesis	54 (1.38 %)	337 (0.95 %)	4.068123e-02	ko00906
Benzoxazinoid biosynthesis	33 (0.84 %)	184 (0.52 %)	4.068123e-02	ko00402
Purine metabolism	168 (4.29 %)	1254 (3.52 %)	4.496028e-02	ko00230

**Table 3:** KEGG pathways related to hormone signaling and intracellular activity

Pathway	Pathway ID	Gene number	Up	Down
Plant hormone signal transduction	ko04075	312	92	220
RNA polymerase	ko03020	135	30	105
ABC transporters	ko02010	57	13	44
Zeatin biosynthesis	ko00908	54	13	41
Brassinosteroid biosynthesis	ko00905	18	1	17
Histidine metabolism	ko00340	10	1	9
Endocytosis	ko04144	42	29	13
Protein processing in endoplasmic reticulum	ko04141	110	69	41
SNARE interactions in vesicular transport	ko04130	19	11	8
Nitrogen metabolism	ko00910	17	4	13
DNA replication	ko03030	19	8	11
Homologous recombination	ko03440	11	3	8
Phagosome	ko04145	36	14	22
Mismatch repair	ko03430	11	4	7
Basal transcription factors	ko03022	8	7	1
Base excision repair	ko03410	10	2	8
Aminoacyl-tRNA biosynthesis	ko00970	18	1	17
RNA degradation	ko03018	30	22	8
Ribosome biogenesis in eukaryotes	ko03008	29	8	21
Protein export	ko03060	6	2	4
mRNA surveillance pathway	ko03015	35	21	14
Ubiquitin mediated proteolysis	ko04120	49	25	24
Nucleotide excision repair	ko03420	15	5	10
RNA transport	ko03013	62	28	34
Proteasome	ko03050	6	3	3
Spliceosome	ko03040	43	29	14
Total		1162 (100 %)	445 (38.30 %)	717 (61.70 %)

To validate the reliability of the expression profiles obtained from RNA-Seq, we selected sixteen DEGs for quantitative real-time PCR (qRT-PCR). Notably, the expressed trends between Q-PCR data and RNA-seq data were consistent generally (Fig. 7, Additional files 3, 4 and 5).

### Expression of sugar metabolism-related genes

**Fig. 6:**
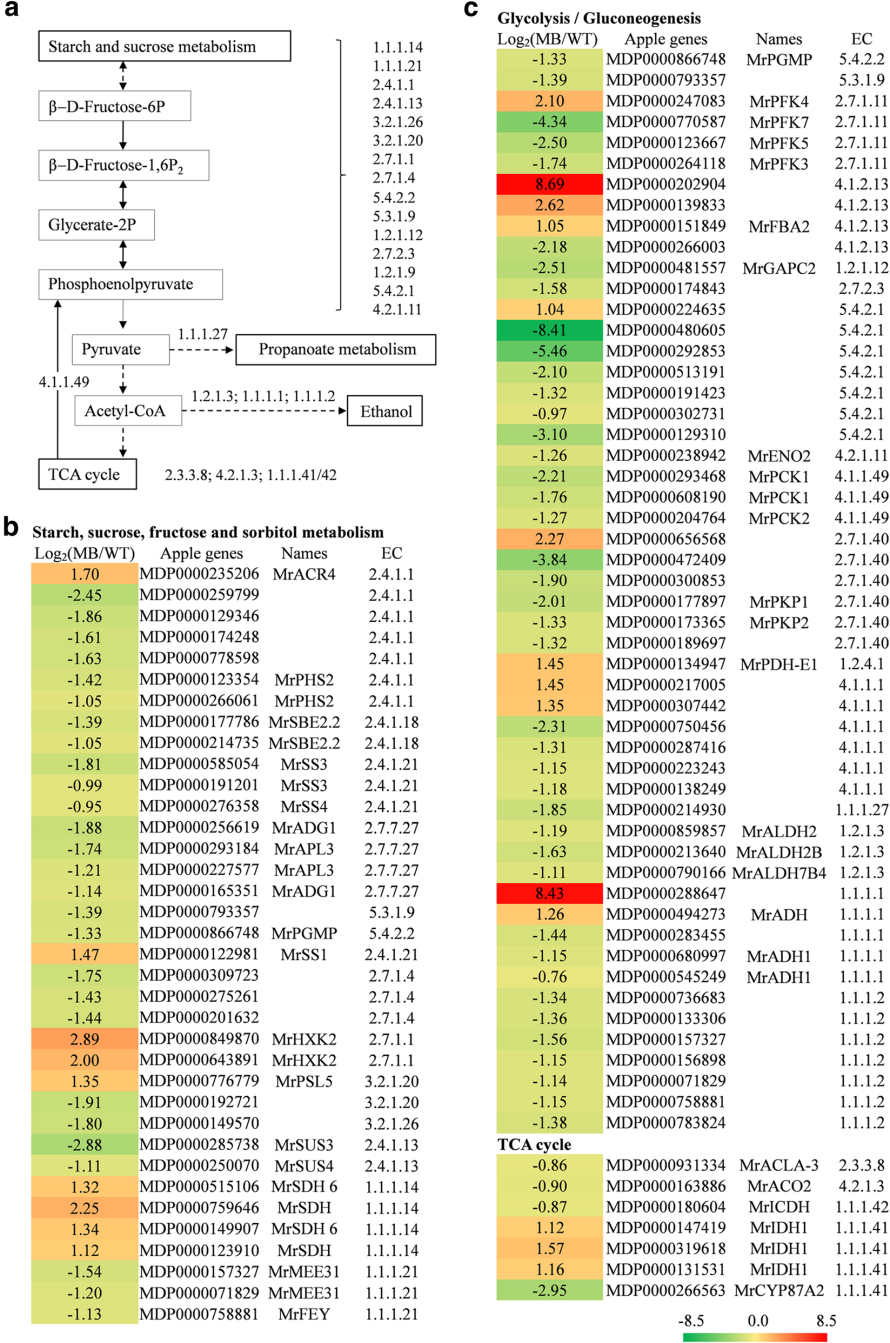
Selected genes related to sugar metabolism from RNA sequencing data. **a** A schematic diagram of sugar metabolism from starch and sucrose metabolism to TCA cycle. Bounding boxes represent metabolic processes or metabolite, and digit represents regulatory enzyme for specific process. Straight arrow indicates transformational direction of metabolite (solid lines, directly; dotted lines, indirectly). **b** Selected genes related to starch, sucrose, fructose and sorbitol metabolism. **c** Selected genes related to glycosis and gluconeogenesis and TCA cycle. Colors indicate the expression values: red indicates up-regulated expression and green indicates down-regulated expression. Data is log base 2 relative to WT and bases on two duplicates

Sugar content and metabolism were correlated with the status of energy supply status at the branching stage. As shown in the list of sugar metabolism-related genes, most genes especially in the process of glycolysis were significantly repressed in roots of MB-bearing rootstock (Fig. 6 and Additional file 3). Sucrose and glucose contents were undifferentiated, however, while fructose content was a slightly elevated in roots of grafted MB (Fig. 2).

**Fig. 7:**
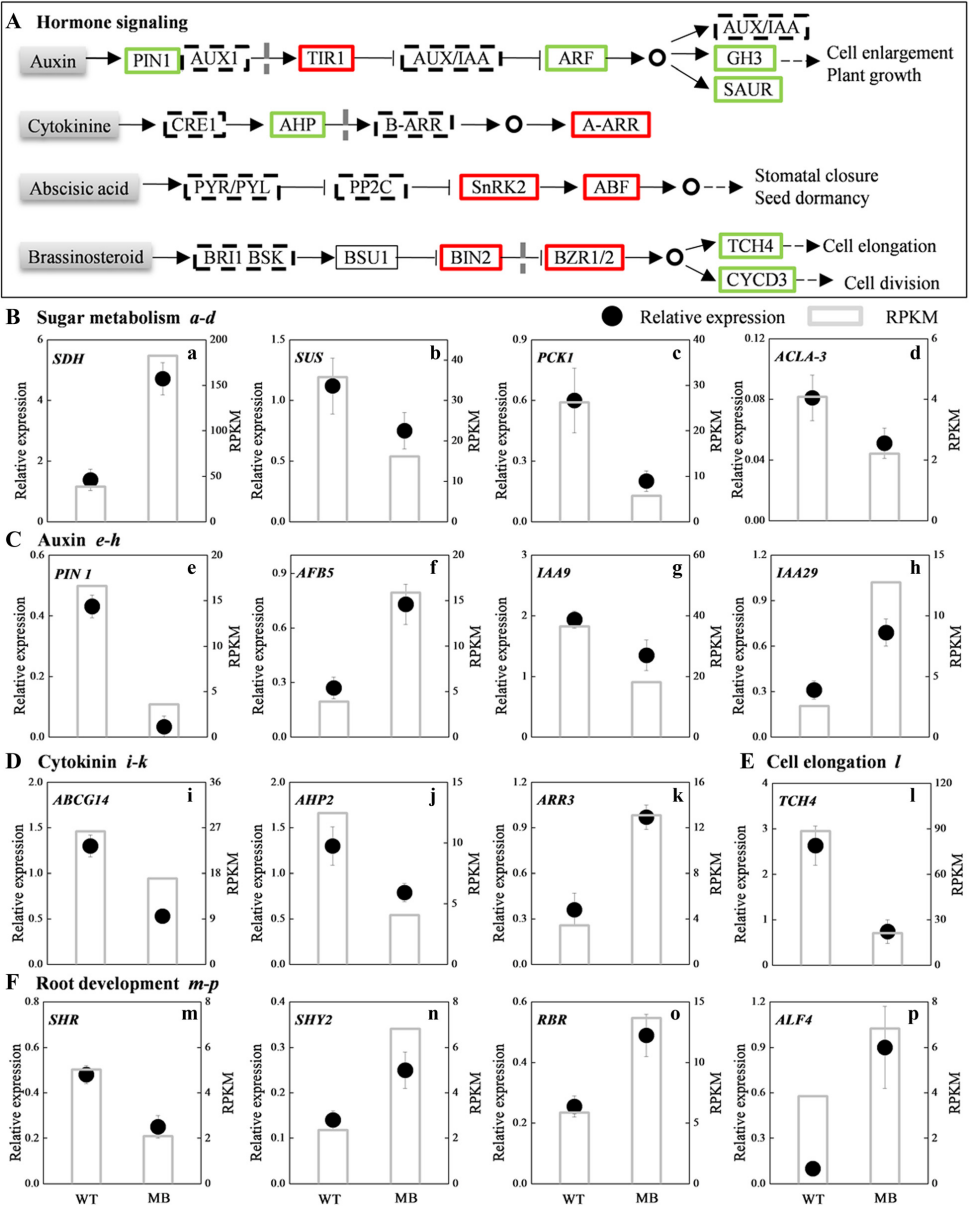
Changes in genes related to hormone signaling pathways, cell elongation and root development detected by DEGs analysis and qRT-PCR identification. **a** DEGs were mapped to plant hormone signal transduction pathways in KEGG database. Up-regulated, down-regulated, up/down-regulated genes and no DEGsare indicated by red, green, dashed and line boxes, respectively. The nucleus is shown behind vertical dashed lines. **b**–**f** QRT-PCR-based identification of sugar metabolism, auxin, cytokinin, cell elongation and root development-related genes. Values are means ± SE (*n* = 3)

Sorbitol is the primary photosynthate, translocated carbohydrate and reserve material in woody Rosaceae species such as apple and pear [43]. NAD^+^-dependent sorbitol dehydrogenase (NAD-SDH; EC 1.1.1.14) catalyzes the transformation of sorbitol to fructose. The four *MrSDHs* identified from the DEG analysis were all up-regulated (Figs. 6b and 7b). In constrast, isomerase and oxidoreductase genes regulating the transformation of sorbitol to glucose, such as *MrMEE31* (*MDP0000275261* and *MDP0000071829*) and *MrFEY* (*MDP0000758881*), were down-regulated.

To carry out sugar metabolism via the glycolytic pathway, fructose-6P functions as an integrated element downstream of glucose and fructose. Fructose-6P is synthesized by aldolase superfamily protein (MDP0000309723), mannose-6-phosphate isomerase type I (MDP0000275261) and an NAD(P)-linked oxidoreductase superfamily protein (MDP0000201632), all of which were significantly repressed in MB-bearing rootstock roots (Fig. 6b). Aconitase 2 (MDP0000180604), isocitrate dehydrogenase (MDP0000163886) and ATP-citrate lyase A-3 (MDP0000931334) in tricarboxylic acid (TCA) cycle were down-regulated. Phosphoenolpyruvate carboxykinase 1 (EC 4.1.1.49), which catalyzes the synthesis of phosphoenolpyruvate that mediates glycolysis and gluconeogenesis, was also down-regulated (Figs. 6c and 7b).

### Expression of hormone signaling-related genes

Selected hormone signaling-related genes encoding receptors and response factors, identified from KEGG pathway analysis, were shown (Fig. 7a, c-d, Additional file 4). The most obvious physiological effect of auxin is the promotion of organ and whole plant growth, which requires auxin transportation and signal transduction. Auxin transporters identified from the apple genome, *MrPIN1* (*MDP0000138035*), two *AUXIN-RESIST-ANT1* (*MrAUX1*; *MDP0000155113*, *MDP0000s749280*) and one *NO VEIN* (*MrNOV*; *MDP0000806699*) were repressed in grafted MB (Fig. 7a, c and Additional file 4), whereas, *MrPIN3* (*MDP0000497581*) and *MrAUX1* (*MDP0000080407*, *MDP0000175425*) were activated. Auxin receptor gene *TRANSPORT INHIBITOR RESPONSE1* (*MrTIR1*; *MDP0000498419*, *MDP0000125975*), *AUXIN F-BOX PROTEIN 5* (*MrAFB5*; *MDP0000809218*, *MDP0000734661*) and *MrSKP2A* (*MDP0000257953*) were activated in MB-bearing rootstock roots, but identified auxin response factors (ARFs) (*MDP0000173151*, *MDP0000123466*, *MDP0000179650*, *MDP0000634433*, *MDP0000412781*, *MDP0000194603*) were repressed (Fig. 7a, c and Additional file 4). *MrGH3* and *small auxin up RNA* (*MrSAUR*), regulating cell enlargement and plant growth downstream of *Mr*ARFs, were down-regulated (Fig. 7a and Additional file 4). *MrTCH4* and *MrCYCD3*, which are downstream of BR signaling and respectively responsible for cell elongation and cell division, were also down-regulated (Fig. 7a, e and Additional file 5).

Most CKs receptors (*MrCRE1s*), which mediate CK-mediated repression of LR initiation [32], were significantly repressed in grafted MB roots (Fig. 7a, d and Additional file 4). The genes encoding histidine-containing phosphotransmitters (AHPs), which are positive regulators of CK signaling, were down-regulated. Some type B response regulators (B-ARR) genes were up-regulated while others were simultaneously down-regulated. *MrARRs* (*MDP0000119750*, *MDP0000250737*), which are suppressors of B-ARRs after the acceptance of a phosphate group from AHPs, were significantly up-regulated [44].

Finally, ABA response factors *MrSnRK2s* and *MrABFs* were up-regulated in roots of grafted MB (Fig. 7a and Additional file 4). These genes are mainly activated by abiotic stress conditions, such as salt stress, water deprivation and osmotic stress [45, 46].

### Expression of genes related to root development, the cell cycle and growth

Several development-related genes operating in root meristem were selected to observe their activities (Fig. 7a, e, f, Additional files 4 and 5). In PRs of grafted MB, Several positive regulatory genes, including *MrSHR* (*MDP0000165587*), *MrAHK3* (*MDP0000181429*), *MrIAA26* (*MDP0000130583*, *MDP0000753736*, *MDP0000164095*), *POLTERGEIST LIKE 1* (*MrPLL1*; *MDP0000256052*) and *TORNADO 2* (*MrTRN2*; *MDP000014257*4), were repressed significantly, whereas some negative regulatory genes, including *WOODEN LEG 1* (*MrWOL*; *MDP0000242242*), *MrTIR1* (*MDP0000125975*, *MDP0000498419*), *MrSHY2* (*MDP0000324919*), *RETINOBLASTOMA-RELATED 1* (*MrRBR*; *MDP0000172418*) and *KIP-RELATED PROTEIN 2* (*MrKRP2*; *MDP0000258414*), were significantly up-regulated (Fig. 7a, f and Additional file 6). *MrALF4* (*MDP0000167283*), a positive regulator during LR formation, was activated in roots of grafted MB (Fig. 7f). Moreover, *LIKE AUXIN RESISTANT 2* (*MrLAX2*; *MDP0000885425*) and *MrALF1* (*MDP0000124971*), which negatively regulate the formation of aberrant LRs, were more than 3-fold down-regulated in roots of grafted MB (Additional file 6). These results were consistent with observed root phenotypes (Fig. 1c and d).

Cells are the basic structural and functional units of organisms. Selected cell cycle-related genes (Fig. 7a and Additional file 5), namely, *MrCYCD1* (*MDP0000809276*, *MDP0000231873*, *MDP0000310564*), *MrCYCD2* (*MDP0000176105*), *MrCYCD3* (*MDP0000286130*, *MDP0000155259*), *GLUCAN SYNTHASE-LIKE 2* (*MrGLS2*; *MDP0000286691*), *K+ TRANSPORTER 1* (*MrKT1*; *MDP0000216786*) and *MrKAC2* (*MDP0000213592*), were significantly repressed in roots of MB-bearing rootstock. Two *MrTCH4* genes (*MDP0000842877*, *MDP0000225088*), which are positively regulated by BR signaling as well as *MrCYCD3*, were down-regulated in roots of grafted MB.

## Discussion

### Scion characteristics affect rootstock sugar metabolism

KEGG biological functions pathway analysis indicated that most genes related to hormone signal transduction, cell transcription and translation activity were down-regulated (Table 3), thus suggesting that root growth and development rates were repressed both at subcellular and transcriptional levels. However, more intensive changes requires further biochemical verification.

Hetero-grafting experiments have demonstrated that some phloem-mobile transcripts target to root tips and modify root architectures [11]. Given the consistent genetic background between WT and MB, any changes in root phenotypes in the same growth environment can be assumed to stem from differences in scion characteristics (Fig. 1). Plant growth and development is dependent on hormonal triggers and metabolic factors, such as photosynthetic efficiency and nutrient uptake [15].

Photoassimilates are the only source of sugar in photosynthetic organisms. Although sorbitol content was dramatically decreased in grafted MB relative to the WT, mainly because of limited photosynthetic ability and transport volume, the contents of sucrose, fructose and glucose in root, required for the glycolytic pathway, were unchanged. The up-regulation of *NAD-SDHs* is only useful for the sorbitol to fructose transition. Genes related to the TCA cycle as well as most glycolysis genes were also significantly repressed in grafted MB (Fig. 6b-c). Recent studies found that sucrose could also serve as a signal prior to hormonal action in apical dominance and bud outgrowth, and that this was positively concentration-dependent [30, 47]. These results suggest that the growth and development of grafted MB suffers, at least metabolically, from limitations to glycolysis and the TCA cycle.

### Regulation of root development and growth by auxin and CK signaling

In plants, the auxin pathway, including polar auxin transport and auxin signal transduction processes, is involved in stages of embryonic and postembryonic root development ranging from hypophysis to meristem initiation, emergence and elongation [48–51].

Auxin has tissue-specific and exerts contradictory activities in roots. At high concentrations, auxin inhibits root elongation. Because grafted WT and MB roots had similar auxin contents, however, reduced auxin levels cannot be responsible for the low rate of root growth observed in MB-bearing rootstock (Fig. 3c). Studies have shown that PIN1 and PIN2 localization directs auxin flow in plants; this is particularly true of downward PIN1-dependent flow, which is positively associated with flow volume and growth rate [52–54]. Consistent with those study findings and the low growth rate of grafted MB roots observed in our study, we discovered that the expressions of *MrPIN1* and related genes such as *MrSHR* were down-regulated in roots grafted MB (Fig. 7a and f). Conversely, however, high auxin and down-regulated expression of *MrPIN1* can help induce division and differentiation of founder cells [55], events that precede the expression of genes needed for LR formation. Although PIN2, PIN4 and PIN7 can specifically change the distribution of auxin in the root elongation zone [56], the expression of their encoding genes was not detected in this study. Finally, up-regulated *MrPIN3* can also increase auxin distribution into pericycle cells to trigger pericycle cell division [57, 58].

Following auxin distribution, auxin signal transduction induces downstream gene expression or crosstalk with other hormone signals. In the present study, the presence of an up-regulated auxin receptors, MrTIR1 and MrAFB5, was consistent with the observed higher auxin content and down-regulation of MrAux/IAA transcriptional repressor proteins (Figs. 3, 7a and Additional file 4). As all the genes influenced by auxin are controlled by the activation or inhibition of MrARFs [59]. Therefore, *ARFs* down-regulation may be ultimately responsible for the low rate of root growth observed in grafted MB.

Like auxin, CKs also play a crucial role in regulating meristem activity [60]. According to a previous study, ZR is the main suppressor of lateral and adventitious root formation [61]. Experimental verification involving CK deficiency and exogenous application indicates that CKs inhibit PR growth and LR initiation within a restricted root meristem region via CRE1/AHK3/B-ARRs signaling [27, 29, 32]. One mode of CK action is the regulation of the expression of genes such as *CRE1*, *SHY2* and *PIN1* as well as cell cycle-related genes [30, 32, 62]. In another mode, which does not involve transcripts, CKs specifically promote PIN1 degradation by disturbing its endocytic recycling in vacuoles, and reduce its abundance on the plasma membrane through an unknown mechanism [29]. In MB-bearing rootstock, root phenotypic changes such as PR length and LR number were accordingly consistent with changes of CK signaling components, such as the up-regulation of *MrSHY2* and *MrA*-*ARRs* (Figs. 1d, f and 7a and Additional file 5). In a study in Arabidopsis, however, exogenous auxin could not rescue the CK-mediated inhibition of LR initiation; in addition, the effect of CKs effect on LR formation was similar between the wild type and auxin mutants harboring in response- and transportation-associated defects [32]. Consequently, CKs and auxin signaling pathways are partially independent during LR initiation; their interaction is more than just a simple antagonism, however, with a balance probably maintained between them in restricted root regions.

### Mediation of multiple signaling by root development-related genes

Specific genes associated with root meristematic activity and differentiation have been identified from root mutants [36]. In Arabidopsis PRs, the balance between cell division and differentiation is coordinated by SHY2, which regulates auxin signaling negatively and CK signaling positively, respectively [28]. *SHY2* expression in grafted MB may have been influenced by CK signaling. As a positive signal of longitudinal growth, *SHR* expression was specific to roots, dependent on root development and growth [38], and highest in roots of grafted WT. Expressions of *MrGH3.9* and *MrTCH4,* responsible for cell elongation or enlargement, were also higher—a response to auxin and BR, respectively (Fig. 7a and Additional file 5).

Overexpression of *RBR* rapidly represses stem cell properties, and delays cell division and differentiation [63, 64]. These changes are similar to the inhibitory effect of KPR2 on CYCD2;1 and cyclin-dependent kinase, which is negatively regulated by auxin [65, 66]. *CYCD2;1* expression is regulated by sucrose, however, which is not responsive to auxin [66]. In our study, *MrKPR2* expression was up-regulated in roots of MB-bearing rootstock with higher auxin content (Fig. 3 and Additional file 6), perhaps because this gene is located downstream of MrARFs.

In contrast to the *alf4* phenotype, the *aberrant lateral root formation-1* (*alf1*) mutant is characterized by an increased number of LRs [39, 67]. Additionally, *ALF4* expression and subcellular locatization of ALF4 are essential to maintain the mitosis of pericycle cells, a process independent of auxin signaling [39]. However, several studies have shown that auxin plays an essential role from LR initiation to LR growth [32, 68]. Given the expression trends observed for *MrALF1* and *MrALF4* in our study, grafted MB should have had a significantly higher number of LRs than grafted WT (Fig. 7f). Consequently, these findings may indicate that the expression of some independent genes for LR development can be repressed by auxin or CK signaling pathways.

### Cell division and differentiation during root growth and development

In roots, active cell division and differentiation primarily occurs in the root meristem zone and may thus regulate the rate of root growth and development [14, 27, 69]. In regard to root growth, the down-regulated expression of cell division- and differentiation- related genes was thus consistent with the root phenotype of grafted MB (Fig. 1d and Additional file 5). The stimulatory effect of auxin on cell division is strongly linked to cell cycle processes. With respect to auxin response, many cell cycle-related genes were in fact up-regulated in roots, whereas cell cycle protein inhibiting factors such as *KRP1*/*2*, were down-regulated [33, 48, 51].

Apart from auxin, the positive role of BR on root growth and development can also be triggered by stimulating the cell cycle in PR meristem [70]. Moreover, exogenously applied BR can promote the expression of auxin response genes and LR development, with these effects inhibited by N-(1-naphthyl) phthalamic acid (NPA) [16, 71]. When exogenous BR is present, LR development is further activated by the addition of exogenous auxin, which may indicate the existence of a synergistic effect between auxin and BR in root development. In our study, however, *brassinazole resistant 1*/*2* (*BZR1*/*2*) genes, which are positive regulators of the BR signalling pathway, were activated in MB-bearing roots (Fig. 7a and Additional file 4). This result may be evidence that auxin signaling genes function downstream of the BR signaling pathway to regulate root cell division.

Although the cell cycle and cell division are insufficient to activate the formation of LRs or buds [34, 72], the processes of cell division, differentiation and enlargement are required for LR initiation, emergence and elongation, even when restricted to pericycle cells [27, 32]. Furthermore, the overall number of LRs was high in grafted MB (Fig. 1f). This finding suggests that pericycle cell division and differentiation were active in roots of grafted MB. Importantly, these results thus obviously indicate that increased rates of cell division and differentiation in MB were mainly limited to root growth. As inferred from the correspondence with root morphology in MB (Fig. 1d), the regulated expression of cell cycle- and growth-related genes may be a direct indicator of root growth.

### Scion influences on root resistance and nitrogen metabolism

ABA plays an important role in plant dormancy and stress resistance [45, 46]. Although previous studies have variously found that ABA has negative or positive effects on root development, its effect is mainly related to auxin pathways [18, 73]. Compared with shoot tips and stems, the ABA content of roots was significantly lower in all grafted seedlings (Fig. 3). Moreover, the undifferentiated concentration of soluble sugars in roots, unlike that of leaves, was insufficient to increase osmotic pressure—a factor playing an important role in abiotic stress response [74, 75]. These results suggest that roots are maintained in a relatively stable environment.

Further analysis revealed that the expressions of ABA response factor genes, which induce dormancy and stress responses, were significantly up-regulated in roots of grafted MB (Fig. 7a and Additional file 7). Moreover, expressions of salicylic acid signal-related genes (*NPR1* and *PR-1*) for disease resistance were significantly repressed in roots of MB (Additional file 7). Concerning root vigor, expressions of nitrogen metabolism related-genes for nitrogen uptake and synthesis of amino acids were repressed (Additional file 7). These results indicate that root vigor was suppressed in grafted MB, possibly leading to limitations on root development and growth.

## Conclusions

In this study, root phenotypes of identical rootstock materials were notably influenced by a more branching mutant, compared to wild type. To gain global insights into the molecular mechanisms responsible for different phenotypes based on consistent genetic backgrounds of scions, RNA-seq of roots was performed to analyze gene expression patterns of development-related biological processes. Specific biological functions analysis indicated that most genes related to hormone signal transduction, cell transcription and translation activity and sugar metabolism were down-regulated. A combined analysis of plant growth dynamics and sugar and hormone contents in roots indicated that scion characteristics can influence rootstock phenotypes, which were mainly regulated by sugar metabolism and auxin and CK signaling pathways. These results may be helpful for further understanding of the mechanisms that cause grafting and internal conditions to influence root growth and development. Moreover, root activity and resistance are also repressed in roots of grafted MB. These results thus imply that the scion characteristics influencing these processes contribute to root growth and development, resistance and activity of rootstock.

## Methods

### Plant material

Scions from wild-type (WT) *M. spectabilis* ‘Bly114’ and a branching mutant (MB) were grafted onto 1-year-old *M. robusta* seedlings. The grafted seedlings were field-cultivated under natural conditions in Yangling (34°52′N, 108°7′E), Shaanxi, China.

For measurements of phytohormone contents and total RNA extraction during the annual growth period when scions presented different branching phenotypes, rootstock roots and scion stems and shoot tips from grafted seedlings were frozen separately in liquid nitrogen and stored at −80 °C until further use.

### Measurement of root phenotypes and photosynthetic parameters

Three intact seedlings each of grafted WT and MB were obtained for root and photosynthetic parameter measurements. Roots were scanned using EPSON EXPRESSION 10000 XL, and images were analyzed using WinRHIZO system (Regent Instruments, Québec, Canada) [76].

A LI-6400 T portable photosynthesis system (Li-Cor, Lincoln NE, USA) was used for in vivo measurements of photosynthetic parameters, including *Pn*, *Gs* and *Ci* on sunny days between 9:00 and 11:00 a.m. Leaves were illuminated with a 6400-02B light source at a saturating incident photosynthetic photon flux density of 1000 μmol m^−2^ s^−1^ from 670-nm red light-emitting diodes with 10 % blue light.

### Measurement of sugar and hormone contents

A total of 0.3 g (dry weight) of roots, stems, leaves and shoot tips from at least three individual seedlings were used for measurements of soluble sugar and determination of hormone contents [77].

To quantify content of ZR, ABA, IAA and GAs contents, 0.2-g fresh tissues samples were prepared for phytohormone extractions, with hormonal analysis and quantification were performed using the enzyme-linked immunosorbent assay (ELISA) technique [78]. After thorough grinding in liquid nitrogen, the samples were extracted overnight with extracting solution at 4 °C. The extracts were collected after centrifugation, passed through a Sep-Pak C_18_ cartridge and dried under N_2_. The residues were dissolved in phosphate buffer. The ELISA for ZR, ABA, IAA and GAs was performed on a 96-well microtitration plate. After adding standard hormone, sample extracts and antibodies, the coated plates were incubated for 40 min at 37 °C. After rinsing four times, 100 μL peroxidase-labeled goat antirabbit immunoglobulin was added to each well and the plate was incubated for 40 min at 37 °C. Colored substrate (o-phenylenediamine) was added to each well, and the reaction was halted by the addition of 3 M H_2_SO_4_. Absorbance at 490 nm was detected using an ELISA spectrophotometer and used to calculate ZR, ABA, IAA and GAs contents [79]. Each sample was measured three times, with three replicates.

### Total RNA isolation

Total RNA was isolated from each sample by a modified method [80], and cDNA was synthesized as previously described [81]. RNA integrity was checked on an agarose gel, with RNA concentrations determined using a Nanodrop1000 spectrophotometer (NanoDrop Technologies, Wilmington, DE, USA).

### RNA-seq and sequencing

Total RNA from roots of grafted with WT and MB was subjected to RNA-seq. After total RNA extraction and DNase I treatment, mRNA was isolated from total RNA using magnetic oligo (dT) bends. The mRNA was mixed with fragmentation buffer and cleaved into short fragments for use as templates for cDNA synthesis. Short fragments were purified, resolved with EB buffer for end reparation and single adenine nucleotide addition and connected with adapters. After agarose gel electrophoresis, suitable fragments were selected as templates for polymerase chain reaction (PCR) amplification. During quality control (QC) steps, an Agilent 2100 Bioanalyzer and an ABI StepOnePlus Real-Time PCR system were used for quantification and qualification of sample libraries. Finally, the constructed libraries were sequenced on an Illumina HiSeq 2000 system (BGI, Shenzhen, China).

### Transcriptome analysis

Primary sequencing data, or raw reads, produced on the Illumina system were filtered into clean reads that were aligned to the apple (*M. domestica*) reference genome (http://www.nature.com/ng/journal/v42/n10/full/ng.654.html) using SOAPaligner/SOAP2 [82].

Then clean reads was used to calculate the mapped reads on the apple genome and to perform coverage analysis (genome mapping rate) [83]. Gene coverage, the percentage of a gene covered by reads, was calculated as the ratio of the base number in a gene covered by unique mapping reads to the total base number of that gene (gene mapping rate).

### Analysis of DEGs

Unigene expression was calculated using the Reads Per kb per Million mapped reads (RPKM) method [84]. The RPKM method eliminated the influence of different gene lengths and sequencing discrepancies on the gene expression calculations. The calculated gene expression could therefore be used to directly compare differences in gene expression between the samples. In case where more than one transcript was obtained for a gene, the longest transcript was used to calculate expression level and coverage. We identified DEGs between WT and MB transcriptomes according to the following criteria [85]: FDR < 0.001, |log_2_Ratio| ≥ 1, and RPKM ≥ 1 at least in one sample.

### GO and KEGG pathway enrichment analyses

DEGs were subjected to GO and KEGG pathway enrichment analyses. Compared with the whole genome background, GO enrichment analysis using DAVID (https://david.ncifcrf.gov/) identified GO terms that were significantly enriched in the list of DEGs and filtered the DEGs corresponding to specific biological functions [86]. KEGG Pathway enrichment analysis in the KEGG Database (http://www.genome.jp/kegg/) was used to identify significantly enriched metabolic or signal transduction pathways in the DEGs [87]. MapMan software was used to display expression profiles at the pathway level [88]. The expression profiles of the metabolic pathways can be viewed by a discrete signal visualized using different colors (blue and red).

### Quantification of gene expression

Specific primers for quantitative real-time PCR (qRT-PCR) were designed using Primer 3 software (Additional file 8). To determine the expression of the target genes, PCR amplifications were performed in a 20-μL containing SYBR Premix Ex Taq II (Tli RNaseH Plus), with 10 μL of 2× SYBR Premix Ex Taq II (Takara, Beijing, China), and 0.8 μL of forward and reverse primers on an iCycler iQ5 (Bio-Rad, USA). The cycling protocol consisted of 95 °C for 180 s, followed by 39 cycles of 95 °C for 15 s, 58 °C for 20 s and 72 °C for 20 s, followed by 39 cycles to construct a melting curve. The *actin* gene was used as an internal control for gene expression normalization. Each reaction was performed in triplicate. The correlation of target genes in expression profiles was measured by qRT-PCR.

### Statistical analysis

Statistical processing of plant phenotype data, sugar and hormone contents and qRT-PCR results was performed in Excel 2007. Differences among means were evaluated by the two-tailed *t*-test with the Statistical Program for Social Science 19 (SPSS, Chicago, IL, USA). Graphs were generated in Excel 2007 and Origin Pro 7.5.

### Availability of data and material

The datasets supporting the conclusions of this article are included within the article and additional files. The dataset of root transcriptomes supporting the conclusions of this article is available in the NCBI Sequence Read Archive repository, the accession is SRR3095691 (http://trace.ncbi.nlm.nih.gov/Traces/sra/sra.cgi?view=run_browser), which will be released at 2017-1-6.

## Additional files

Additional file 1:
**Photosynthetic parameters of WT and MB grafted apple leaves.** Comparison of photosynthetic parameters, including net photosynthetic rate (*Pn*), stomatal conductance (*Gs*) and intercellular CO_2_ concentration (*Ci*), in WT and MB leaves. Values are means ± SE (*n* = 10). Significant differences (**P* < 0.05 and ***P* < 0.01) are based on Student’s *t*-tests. (DOC 111 kb) Additional file 2:
**Clusters of annotated GO terms in the biological process category enriched in Up-regulated (A) and Down-regulated (B) between roots of grafted WT and MB apple.** (DOC 2423 kb) Additional file 3:
**Selected genes related to sugar metabolism.** (DOC 93 kb) Additional file 4:
**Differentially expressed genes related to hormone signaling.** (DOC 194 kb) Additional file 5:
**Selected root development-related genes from RNA sequencing data.** (DOC 46 kb) Additional file 6:
**Selected differentially expressed genes related to cell division, differentiation and growth.** (DOC 39 kb) Additional file 7:
**Selected differentially expressed genes related to salicylic and jasmonic acid signaling and nitrogen metabolism.** (DOC 48 kb) Additional file 8:
**Gene-specific primers used for quantitative real-time PCR.** (DOC 37 kb)

## Abbreviations

ABA: abscisic acid
CK: cytokinin
LR: lateral root
MB: more branching mutant
PR: primary root
WT: wild type
