# Supplementary material for: Transcriptome analysis reveals the effects of sugar metabolism and auxin and cytokinin signaling pathways on root growth and development of grafted apple
Source: BMC Genomics. 2016 Feb 29;17:150. doi: 10.1186/s12864-016-2484-x (PMC4770530; doi:10.1186/s12864-016-2484-x)
Supplement: Additional file 1: — Photosynthetic parameters of WT and MB grafted apple leaves. Comparison of photosynthetic parameters, including net photosynthetic rate (Pn), stomatal conductance (Gs) and intercellular CO2 concentration (Ci), in WT and MB leaves. Values are means ± SE (n = 10). Significant differences (*P < 0.05 and **P < 0.01) are based on Student’s t-tests. (DOC 111 kb) [file 12864_2016_2484_MOESM1_ESM.doc]

Additional file 1: Photosynthetic parameters of WT and MB grafted apple leaves.
