# Supplementary material for: Transcriptome analysis reveals the effects of sugar metabolism and auxin and cytokinin signaling pathways on root growth and development of grafted apple
Source: BMC Genomics. 2016 Feb 29;17:150. doi: 10.1186/s12864-016-2484-x (PMC4770530; doi:10.1186/s12864-016-2484-x)
Supplement: Additional file 2: — Clusters of annotated GO terms in the biological process category enriched in Up-regulated (A) and Down-regulated (B) between roots of grafted WT and MB apple. (DOC 2423 kb) [file 12864_2016_2484_MOESM2_ESM.doc]

Additional file 2: Clusters of annotated GO terms in the biological process category enriched in Up-regulated (A) and Down-regulated (B) between roots of grafted WT and MB apple. DEGs were classified into specific biological process categories using DAVID with high classification stringency (P < 0.05). The horizontal ordinate represents the number of genes in the category.

**
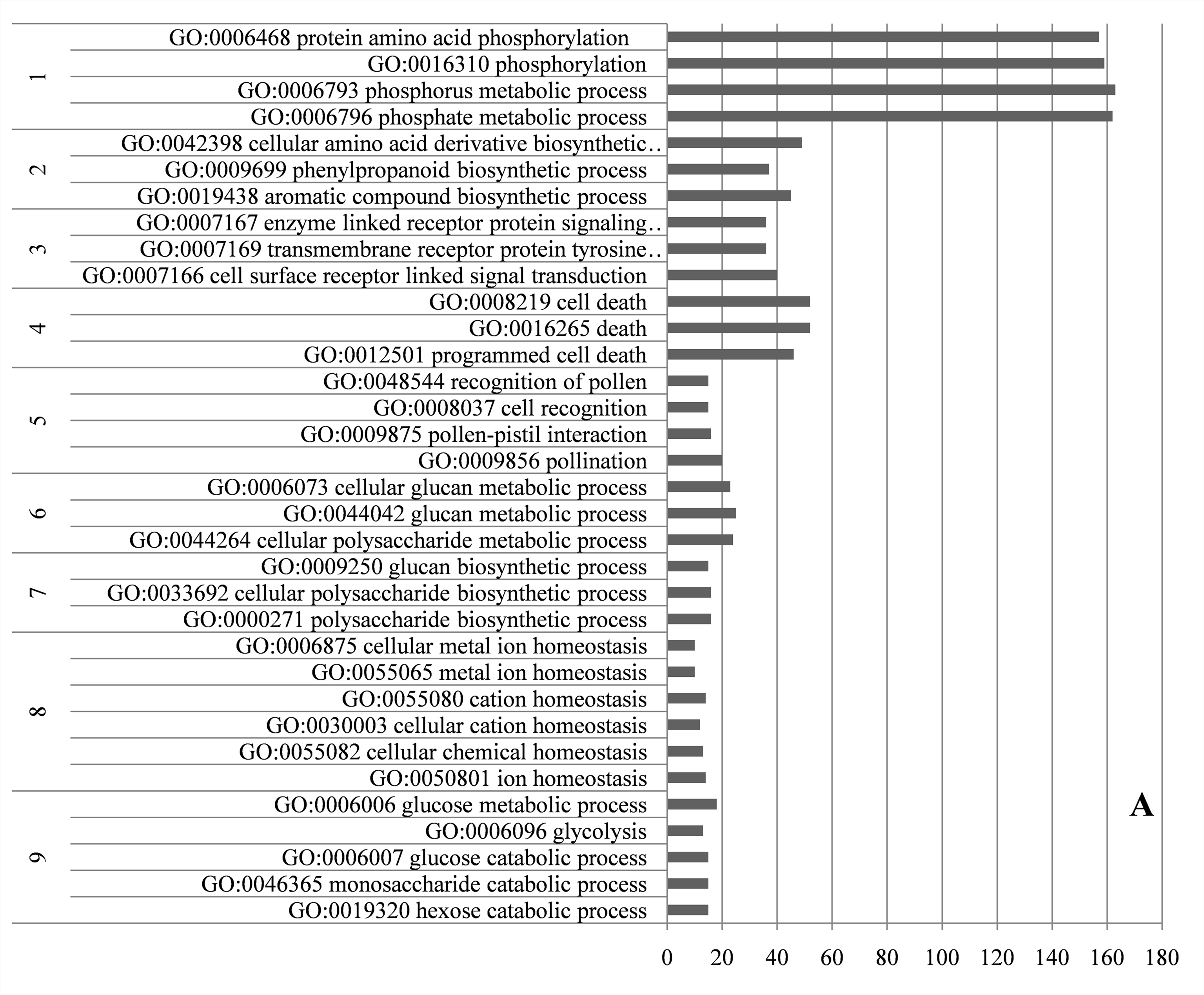
**

**
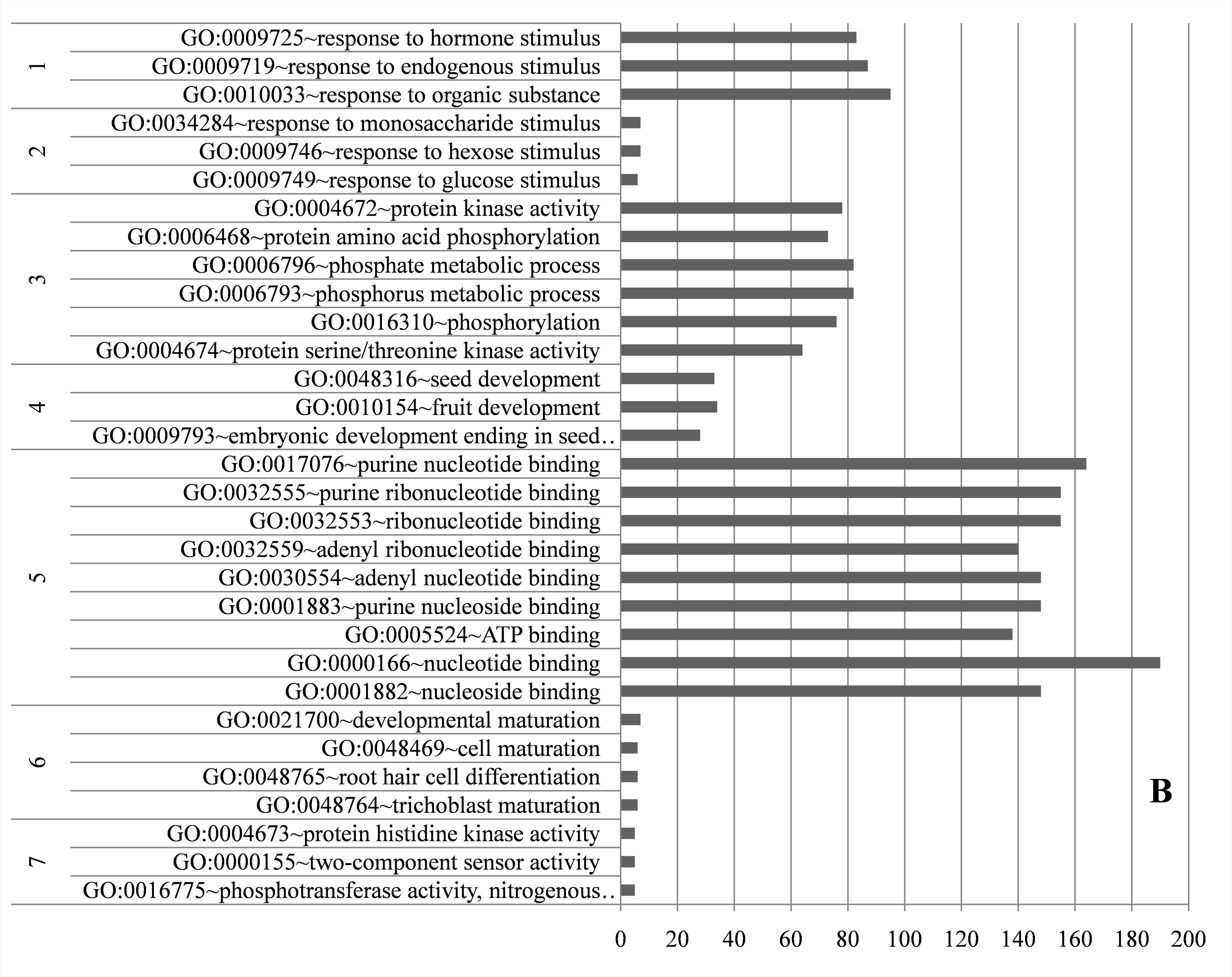
**
