# Supplementary material for: Transcriptome analysis reveals the effects of sugar metabolism and auxin and cytokinin signaling pathways on root growth and development of grafted apple
Source: BMC Genomics. 2016 Feb 29;17:150. doi: 10.1186/s12864-016-2484-x (PMC4770530; doi:10.1186/s12864-016-2484-x)
Supplement: Additional file 3: — Selected genes related to sugar metabolism. (DOC 93 kb) [file 12864_2016_2484_MOESM3_ESM.doc]

**Additional file 3 Selected genes related to sugar metabolism from RNA sequencing data**

| **Apple genes Identification** | **Arabidopsis Homolog** | **Names** | **Annotation** |
| --- | --- | --- | --- |
| **Starch and sucrose metabolism** | |  |  |
| MDP0000849870 | AT2G19860.1 | HXK2 | hexokinase 2 |
| MDP0000643891 | AT2G19860.1 | HXK2 | hexokinase 2 |
| MDP0000235206 | AT1G69040.2 | ACR4 | ACT domain repeat 4 |
| MDP0000776779 | AT5G63840.1 | PSL5 | Glycosyl hydrolases family 31 protein |
| MDP0000175027 | AT3G16850.1 |  | Pectin lyase-like superfamily protein |
| MDP0000249275 | AT4G33440.1 |  | Pectin lyase-like superfamily protein |
| MDP0000582836 | AT3G14040.1 |  | Pectin lyase-like superfamily protein |
| MDP0000251956 | AT3G61490.1 |  | Pectin lyase-like superfamily protein |
| MDP0000296416 | AT1G48100.1 |  | Pectin lyase-like superfamily protein |
| MDP0000285738 | AT4G02280.1 | SUS3 | sucrose synthase 3 |
| MDP0000259799 | AT3G29320.1 |  | Glycosyl transferase, family 35 |
| MDP0000192721 | AT5G11720.1 |  | Glycosyl hydrolases family 31 protein |
| MDP0000610678 | AT4G23500.1 |  | Pectin lyase-like superfamily protein |
| MDP0000250896 | AT3G54090.1 | FLN1 | fructokinase-like 1 |
| MDP0000470297 | AT1G48100.1 |  | Pectin lyase-like superfamily protein |
| MDP0000129346 | AT3G29320.1 |  | Glycosyl transferase, family 35 |
| MDP0000256619 | AT5G48300.1 | ADG1 | ADP glucose pyrophosphorylase 1 |
| MDP0000149570 | AT1G62660.1 |  | Glycosyl hydrolases family 32 protein |
| MDP0000209148 | AT1G23460.1 |  | Pectin lyase-like superfamily protein |
| MDP0000293184 | AT4G39210.1 | APL3 | Glucose-1-phosphate adenylyltransferase family protein |
| MDP0000309723 | AT1G66430.1 |  | pfkB-like carbohydrate kinase family protein |
| MDP0000174248 | AT3G29320.1 |  | Glycosyl transferase, family 35 |
| MDP0000275261 | AT1G66430.1 |  | pfkB-like carbohydrate kinase family protein |
| MDP0000201632 | AT1G19600.1 |  | pfkB-like carbohydrate kinase family protein |
| MDP0000793357 | AT5G42740.1 |  | Sugar isomerase (SIS) family protein |
| MDP0000168416 | AT3G57510.1 | ADPG1 | Pectin lyase-like superfamily protein |
| MDP0000778598 | AT3G29320.1 |  | Glycosyl transferase, family 35 |
| MDP0000177786 | AT5G03650.1 | SBE2.2 | starch branching enzyme 2.2 |
| MDP0000123354 | AT3G46970.1 | PHS2 | alpha-glucan phosphorylase 2 |
| MDP0000266061 | AT3G46970.1 | PHS2 | alpha-glucan phosphorylase 2 |
| MDP0000866748 | AT5G51820.1 | PGM | phosphoglucomutase |
| MDP0000130769 | AT3G62110.1 |  | Pectin lyase-like superfamily protein |
| MDP0000227577 | AT4G39210.1 | APL3 | Glucose-1-phosphate adenylyltransferase family protein |
| MDP0000165351 | AT5G48300.1 | ADG1 | ADP glucose pyrophosphorylase 1 |
| MDP0000250070 | AT3G43190.1 | SUS4 | sucrose synthase 4 |
| MDP0000581832 | AT4G23820.1 |  | Pectin lyase-like superfamily protein |
| MDP0000214735 | AT5G03650.1 | SBE2.2 | starch branching enzyme 2.2 |
| **Sorbitol dehydrogenase** | |  |  |
| MDP0000515106 | AT5G51970.1 | ATSDH 6 | GroES-like zinc-binding alcohol dehydrogenase family protein |
| MDP0000759646 | AT5G51970.1 | ATSDH | GroES-like zinc-binding alcohol dehydrogenase family protein |
| MDP0000149907 | AT5G51970.1 | ATSDH 6 | GroES-like zinc-binding alcohol dehydrogenase family protein |
| MDP0000123910 | AT5G51970.1 | ATSDH | GroES-like zinc-binding alcohol dehydrogenase family protein |
| **Fructose metabolism** | |  |  |
| MDP0000139833 | AT2G19860.1 | HXK2 | hexokinase 2 |
| MDP0000247083 | AT2G19860.1 | HXK2 | hexokinase 2 |
| MDP0000151849 | AT5G50130.1 |  | NAD(P)-binding Rossmann-fold superfamily protein |
| MDP0000926788 | AT5G51970.1 |  | GroES-like zinc-binding alcohol dehydrogenase family protein |
| MDP0000515106 | AT5G51970.1 |  | GroES-like zinc-binding alcohol dehydrogenase family protein |
| MDP0000123910 | AT5G51970.1 |  | GroES-like zinc-binding alcohol dehydrogenase family protein |
| MDP0000250896 | AT5G56630.1 | PFK7 | phosphofructokinase 7 |
| MDP0000309723 | AT2G36460.1 |  | Aldolase superfamily protein |
| MDP0000275261 | AT3G02570.1 | MEE31 | Mannose-6-phosphate isomerase, type I |
| MDP0000201632 | AT1G59960.1 |  | NAD(P)-linked oxidoreductase superfamily protein |
| MDP0000071829 | AT3G02570.1 | MEE31 | Mannose-6-phosphate isomerase, type I |
| MDP0000271872 | AT3G02570.1 | MEE31 | Mannose-6-phosphate isomerase, type I |
| MDP0000758881 | AT4G27760.1 | FEY | NAD(P)-binding Rossmann-fold superfamily protein |
| MDP0000293468 | AT4G37870.1 | PCK1 | phosphoenolpyruvate carboxykinase 1 |
| MDP0000608190 | AT4G37870.1 | PCK1 | phosphoenolpyruvate carboxykinase 1 |
| MDP0000204764 | AT5G65690.1 | PCK2 | phosphoenolpyruvate carboxykinase 2 |
| MDP0000217005 | AT5G01320.1 |  | Thiamine pyrophosphate dependent pyruvate decarboxylase family protein |
| MDP0000307442 | AT5G01320.1 |  | Thiamine pyrophosphate dependent pyruvate decarboxylase family protein |
| MDP0000750456 | AT4G33070.1 |  | Thiamine pyrophosphate dependent pyruvate decarboxylase family protein |
| MDP0000287416 | AT4G33070.1 |  | Thiamine pyrophosphate dependent pyruvate decarboxylase family protein |
| MDP0000223243 | AT4G33070.1 |  | Thiamine pyrophosphate dependent pyruvate decarboxylase family protein |
| MDP0000138249 | AT4G33070.1 |  | Thiamine pyrophosphate dependent pyruvate decarboxylase family protein |
| MDP0000214930 | AT4G17260.1 |  | Lactate/malate dehydrogenase family protein |
| MDP0000859857 | AT3G48000.1 | ALDH2 | aldehyde dehydrogenase 2B4 |
| MDP0000213640 | AT1G23800.1 | ALDH2B | aldehyde dehydrogenase 2B7 |
| MDP0000790166 | AT1G54100.1 | ALDH7B4 | aldehyde dehydrogenase 7B4 |
| MDP0000288647 | AT5G48370.1 |  | Thioesterase/thiol ester dehydrase-isomerase superfamily protein |
| MDP0000494273 | AT1G77120.1 | ADH | alcohol dehydrogenase 1 |
| MDP0000283455 | AT5G42250.1 |  | Zinc-binding alcohol dehydrogenase family protein |
| MDP0000680997 | AT1G77120.1 | ADH | alcohol dehydrogenase 1 |
| MDP0000545249 | AT1G77120.1 | ADH | alcohol dehydrogenase 1 |
| MDP0000736683 | AT1G59960.1 |  | NAD(P)-linked oxidoreductase superfamily protein |
| MDP0000133306 | AT2G21250.1 |  | NAD(P)-linked oxidoreductase superfamily protein |
| MDP0000157327 | AT2G21260.1 |  | NAD(P)-linked oxidoreductase superfamily protein |
| MDP0000156898 | AT2G21250.1 |  | NAD(P)-linked oxidoreductase superfamily protein |
| MDP0000071829 | AT2G21250.1 |  | NAD(P)-linked oxidoreductase superfamily protein |
| MDP0000758881 | AT2G21250.1 |  | NAD(P)-linked oxidoreductase superfamily protein |
| MDP0000783824 | AT2G21250.1 |  | NAD(P)-linked oxidoreductase superfamily protein |
| **TCA cycle** |  |  |  |
| MDP0000931334 | AT1G09430.1 | ACLA-3 | ATP-citrate lyase A-3 |
| MDP0000163886 | AT4G26970.1 | ACO2 | aconitase 2 |
| MDP0000180604 | AT1G54340.1 | ICDH | isocitrate dehydrogenase |
| MDP0000147419 | AT4G35260.1 | IDH1 | isocitrate dehydrogenase 1 |
| MDP0000319618 | AT4G35260.1 | IDH1 | isocitrate dehydrogenase 1 |
| MDP0000131531 | AT4G35260.1 | IDH1 | isocitrate dehydrogenase 1 |
| MDP0000266563 | AT1G12740.2 | CYP87A2 | cytochrome P450, family 87, subfamily A, polypeptide 2 |
