# Supplementary material for: Transcriptome analysis reveals the effects of sugar metabolism and auxin and cytokinin signaling pathways on root growth and development of grafted apple
Source: BMC Genomics. 2016 Feb 29;17:150. doi: 10.1186/s12864-016-2484-x (PMC4770530; doi:10.1186/s12864-016-2484-x)
Supplement: Additional file 4: — Differentially expressed genes related to hormone signaling. (DOC 194 kb) [file 12864_2016_2484_MOESM4_ESM.doc]

**Additional file 4 Differentially expressed genes related to hormone** signaling

| **Apple genes Identification** | **Arabidopsis Homolog** | **Names** | **Cluster** | **Annotation** | **log_2_(MB /WT)** |
| --- | --- | --- | --- | --- | --- |
| **Auxin signaling** |  |  |  |  |  |
| MDP0000138035 | AT1G73590.1 | PIN1 |  | Auxin efflux carrier family protein | -2.22 |
| MDP0000497581 | AT1G70940.1 | PIN3 |  | Auxin efflux carrier family protein | 2.18 |
| MDP0000806699 | AT4G13750.1 | NOV |  | Histidine kinase-, DNA gyrase B-, and HSP90-like ATPase family protein | -1.26 |
| MDP0000138851 | AT1G48910.1 | YUC10 |  | Flavin-containing monooxygenase family protein | 1.34 |
| MDP0000456476 | AT5G65670 | IAA9 |  | indole-3-acetic acid inducible 9 | -1.01 |
| MDP0000280440 | AT1G08230.2 | GAT1 | AUX1 | Transmembrane amino acid transporter family protein | 1.66 |
| MDP0000498419 | AT3G62980.1 | TIR1 | TIR1 | F-box/RNI-like superfamily protein | 2.69 |
| MDP0000125975 | AT3G62980.1 | TIR1 | TIR1 | F-box/RNI-like superfamily protein | 1.38 |
| MDP0000809218 | AT5G49980.1 | AFB5 | TIR1 | auxin F-box protein 5 | 2.02 |
| MDP0000734661 | AT5G49980.1 | AFB5 | TIR1 | auxin F-box protein 5 | 1.96 |
| MDP0000135966 | AT5G49980.1 | AFB5 | TIR1 | auxin F-box protein 5 | 1.22 |
| MDP0000257953 | AT1G21410.1 | SKP2A |  | F-box/RNI-like superfamily protein | 1.00 |
| MDP0000173151 | AT2G33860.1 | ARF3 | ARF | auxin-responsive factor AUX/IAA-related | -2.03 |
| MDP0000123466 | AT5G62000.1 | ARF2 | ARF | auxin response factor 2 | -1.21 |
| MDP0000179650 | AT2G33860.1 | ARF3 | ARF | Transcriptional factor B3 family protein / auxin-responsive factor AUX/IAA-related | -1.03 |
| MDP0000634433 | AT4G23980.1 | ARF9 | ARF | auxin response factor 9 | -1.12 |
| MDP0000412781 | AT5G60450.1 | ARF4 | ARF | auxin response factor 4 | -1.12 |
| MDP0000194603 | AT3G61830.1 | ARF18 | ARF | auxin response factor 18 | -1.03 |
| MDP0000568498 | AT2G47750.1 | GH3.9 | GH3 | putative indole-3-acetic acid-amido synthetase | -1.71 |
| MDP0000786650 | AT2G46370.4 | JAR1 | GH3 | Auxin-responsive GH3 family protein | -1.46 |
| MDP0000209432 | AT2G14960.1 | GH3.1 | GH3 | Auxin-responsive GH3 family protein | -1.28 |
| MDP0000153382 | AT2G24400.1 |  | SUAR | SAUR-like auxin-responsive protein family | -3.48 |
| MDP0000543718 | AT4G32280.1 | IAA29 | AUX/IAA | indole-3-acetic acid inducible 29 | 2.55 |
| MDP0000324919 | AT1G04240.1 | IAA3 | AUX/IAA | AUX/IAA transcriptional regulator family protein | 1.55 |
| MDP0000123816 | AT1G04250.1 | AXR3 | AUX/IAA | AUX/IAA transcriptional regulator family protein | 1.45 |
| MDP0000253285 | AT1G04250.1 | AXR3 | AUX/IAA | AUX/IAA transcriptional regulator family protein | 1.37 |
| MDP0000146848 | AT5G57420.1 | IAA33 | AUX/IAA | indole-3-acetic acid inducible 33 | -2.01 |
| MDP0000090281 | AT2G22670.4 | IAA8 | AUX/IAA | indoleacetic acid-induced protein 8 | -1.44 |
| MDP0000246204 | AT2G22670.4 | IAA8 | AUX/IAA | indoleacetic acid-induced protein 8 | -1.77 |
| MDP0000157035 | AT4G32280.1 | IAA29 | AUX/IAA | indole-3-acetic acid inducible 29 | -1.97 |
| MDP0000753736 | AT3G16500.1 | PAP1 | AUX/IAA | phytochrome-associated protein 1 | -1.74 |
| MDP0000361838 | AT4G29080.1 | PAP2 | AUX/IAA | phytochrome-associated protein 2 | -1.41 |
| MDP0000174664 | AT4G29080.1 | PAP2 | AUX/IAA | phytochrome-associated protein 2 | -1.24 |
| **Cytokinin signaling** | |  |  |  |  |
| MDP0000759591 | AT5G55180.2 |  | CRE1 | O-Glycosyl hydrolases family 17 protein | 3.44 |
| MDP0000295562 | AT5G55180.2 |  | CRE1 | O-Glycosyl hydrolases family 17 protein | 2.56 |
| MDP0000323490 | AT1G18650.1 | PDCB3 | CRE1 | plasmodesmata callose-binding protein 3 | 1.89 |
| MDP0000242242 | AT2G01830.1 | AHK4 | CRE1 | CHASE domain containing histidine kinase protein | 1.46 |
| MDP0000181429 | AT1G27320.1 | AHK3 | CRE1 | histidine kinase 3 | -9.72 |
| MDP0000137803 | AT1G27320.1 | AHK3 | CRE1 | histidine kinase 3 | -10.99 |
| MDP0000659407 | AT1G27320.1 | AHK3 | CRE1 | histidine kinase 3 | -7.67 |
| MDP0000660874 | AT2G05790.1 |  | CRE1 | O-Glycosyl hydrolases family 17 protein | -2.24 |
| MDP0000171994 | AT5G67460.1 |  | CRE1 | O-Glycosyl hydrolases family 17 protein | -1.66 |
| MDP0000321326 | AT2G05790.1 |  | CRE1 | O-Glycosyl hydrolases family 17 protein | -1.52 |
| MDP0000630545 | AT5G56590.1 |  | CRE1 | O-Glycosyl hydrolases family 17 protein | -1.38 |
| MDP0000321387 | AT4G26830.1 |  | CRE1 | O-Glycosyl hydrolases family 17 protein | -1.34 |
| MDP0000146863 | AT4G34480.1 |  | CRE1 | O-Glycosyl hydrolases family 17 protein | -1.44 |
| MDP0000289320 | AT1G18650.1 | PDCB3 | CRE1 | plasmodesmata callose-binding protein 3 | -1.33 |
| MDP0000235663 | AT3G07320.1 |  | CRE1 | O-Glycosyl hydrolases family 17 protein | -0.91 |
| MDP0000754160 | AT5G24318.1 |  | CRE1 | O-Glycosyl hydrolases family 17 protein | -0.99 |
| MDP0000317420 | AT3G16360.2 | AHP4 | AHP | HPT phosphotransmitter 4 | -4.44 |
| MDP0000216664 | AT3G21510.1 | AHP1 | AHP | histidine-containing phosphotransmitter 1 | -2.60 |
| MDP0000753736 | AT3G16500.1 | PAP1 | AHP | phytochrome-associated protein 1 | -1.74 |
| MDP0000231522 | AT3G29350.1 | AHP2 | AHP | histidine-containing phosphotransmitter 2 | -1.65 |
| MDP0000123837 | AT3G29350.1 | AHP2 | AHP | histidine-containing phosphotransmitter 2 | -1.67 |
| MDP0000186518 | AT5G39340.1 | AHP3 | AHP | histidine-containing phosphotransmitter 3 | -2.62 |
| MDP0000669078 | AT3G46640.1 | LUX | B-ARR | Homeodomain-like superfamily protein | 1.64 |
| MDP0000124301 | AT4G16110.1 | ARR2 | B-ARR | response regulator 2 | 1.06 |
| MDP0000773970 | AT2G01060.1 |  | B-ARR | myb-like HTH transcriptional regulator family protein | 1.13 |
| MDP0000265555 | AT1G49560.1 |  | B-ARR | Homeodomain-like superfamily protein | 1.15 |
| MDP0000809773 | AT3G24120.1 |  | B-ARR | Homeodomain-like superfamily protein | -1.62 |
| MDP0000526864 | AT2G20570.1 | GLK1 | B-ARR | GBF\'s pro-rich region-interacting factor 1 | -1.56 |
| MDP0000128135 | AT2G03500.1 |  | B-ARR | Homeodomain-like superfamily protein | -1.16 |
| MDP0000182154 | AT3G13040.1 |  | B-ARR | myb-like HTH transcriptional regulator family protein | -1.32 |
| MDP0000119750 | AT3G57040.1 | ARR9 | A-ARR | response regulator 9 | 1.81 |
| MDP0000250737 | AT1G59940.1 | ARR3 | A-ARR | response regulator 3 | 1.88 |
| **Abscisic acid signaling** | |  |  |  |  |
| MDP0000154764 | AT5G51990.1 | CBF4 |  | C-repeat-binding factor 4 | -0.98 |
| MDP0000192034 | AT3G19270.1 | CYP707A4 |  | cytochrome P450, family 707, subfamily A, polypeptide 4 | 1.40 |
| MDP0000407613 | AT3G23250 | MYB15 |  | myb domain protein 15 | -1.50 |
| MDP0000916879 | AT5G10720.1 | AHK5 |  | histidine kinase 5 | 1.11 |
| MDP0000586302 | AT5G11260.1 | HY5 |  | Basic-leucine zipper (bZIP) transcription factor family protein | 4.90 |
| MDP0000912146 | AT1G15100.1 | RHA2A |  | RING-H2 finger A2A | -4.68 |
| MDP0000270731 | AT2G38310.1 | PYL4 | PYR/PYL | PYR1-like 4 | 3.23 |
| MDP0000284624 | AT1G01360.1 | PYL9 | PYR/PYL | regulatory component of ABA receptor 1 | 1.20 |
| MDP0000420318 | AT4G17870.1 | PYR1 | PYR/PYL | Polyketide cyclase/dehydrase and lipid transport superfamily protein | -0.93 |
| MDP0000647467 | AT1G72770.1 | HAB1 | PP2C | homology to ABI1 | 1.28 |
| MDP0000690444 | AT3G17090.1 |  | PP2C | Protein phosphatase 2C family protein | 0.86 |
| MDP0000296566 | AT2G29380.1 | HAI3 | PP2C | highly ABA-induced PP2C gene 3 | 1.19 |
| MDP0000134377 | AT3G12620.1 |  | PP2C | Protein phosphatase 2C family protein | 1.13 |
| MDP0000731537 | AT3G12620.1 |  | PP2C | Protein phosphatase 2C family protein | -2.12 |
| MDP0000628470 | AT2G40180.1 | PP2C5 | PP2C | phosphatase 2C5 | -1.77 |
| MDP0000893203 | AT1G72770.1 | HAB1 | PP2C | homology to ABI1 | -0.66 |
| MDP0000378947 | AT2G30360.1 | CIPK11 | SnRK2 | SOS3-interacting protein 4 | 2.16 |
| MDP0000189269 | AT4G33950.1 | OST1 | SnRK2 | Protein kinase superfamily protein | 1.24 |
| MDP0000129203 | AT2G40620.1 |  | ABF | Basic-leucine zipper (bZIP) transcription factor family protein | 5.04 |
| MDP0000586302 | AT5G11260.1 | HY5 | ABF | Basic-leucine zipper (bZIP) transcription factor family protein | 4.90 |
| MDP0000205823 | AT1G75390.1 | bZIP44 | ABF | basic leucine-zipper 44 | 1.88 |
| MDP0000261154 | AT4G37730.1 | bZIP7 | ABF | basic leucine-zipper 7 | 1.27 |
| MDP0000296303 | AT1G45249.1 | ABF2 | ABF | abscisic acid responsive elements-binding factor 2 | 1.36 |
| **Brassinosteroid signaling** | |  |  |  |  |
| MDP0000174738 | AT5G05850.1 | PIRL1 | BRI1 | plant intracellular ras group-related LRR 1 | 7.35 |
| MDP0000180713 | AT4G08850.1 |  | BRI1 | Leucine-rich repeat receptor-like protein kinase family protein | 2.68 |
| MDP0000391018 | AT4G03230.1 |  | BRI1 | S-locus lectin protein kinase family protein | 1.34 |
| MDP0000199107 | AT5G43020.1 |  | BRI1 | Leucine-rich repeat protein kinase family protein | 3.20 |
| MDP0000497333 | AT4G08850.1 |  | BRI1 | Leucine-rich repeat receptor-like protein kinase family protein | 1.66 |
| MDP0000196035 | AT1G29750.2 | RKF1 | BRI1 | receptor-like kinase in flowers 1 | 1.80 |
| MDP0000713958 | AT2G34930.1 |  | BRI1 | disease resistance family protein / LRR family protein | 1.45 |
| MDP0000237989 | AT5G07620.1 |  | BRI1 | Protein kinase superfamily protein | 1.35 |
| MDP0000250798 | AT1G07390.3 | RLP1 | BRI1 | receptor like protein 1 | 2.00 |
| MDP0000490329 | AT2G34930.1 |  | BRI1 | disease resistance family protein / LRR family protein | 1.03 |
| MDP0000657568 | AT5G05850.1 | PIRL1 | BRI1 | plant intracellular ras group-related LRR 1 | 0.98 |
| MDP0000157003 | AT4G39400.1 | BRI1 | BRI1 | Leucine-rich receptor-like protein kinase family protein | 1.06 |
| MDP0000574604 | AT2G26730.1 |  | BRI1 | Leucine-rich repeat protein kinase family protein | 0.70 |
| MDP0000120329 | AT1G35710.1 |  | BRI1 | Protein kinase family protein with leucine-rich repeat domain | -8.67 |
| MDP0000277413 | AT4G39400.1 | BRI1 | BRI1 | Leucine-rich receptor-like protein kinase family protein | -7.76 |
| MDP0000114901 | AT3G47570.1 |  | BRI1 | Leucine-rich repeat protein kinase family protein | -5.18 |
| MDP0000543726 | AT2G34930.1 |  | BRI1 | disease resistance family protein / LRR family protein | -6.72 |
| MDP0000719295 | AT3G47580.1 |  | BRI1 | Leucine-rich repeat protein kinase family protein | -9.52 |
| MDP0000203539 | AT2G34930.1 |  | BRI1 | disease resistance family protein / LRR family protein | -5.21 |
| MDP0000184398 | AT4G08850.1 |  | BRI1 | Leucine-rich repeat receptor-like protein kinase family protein | -8.99 |
| MDP0000588587 | AT3G47090.1 |  | BRI1 | Leucine-rich repeat protein kinase family protein | -6.15 |
| MDP0000251336 | AT1G72300.1 |  | BRI1 | Leucine-rich receptor-like protein kinase family protein | -5.99 |
| MDP0000312097 | AT4G21380.1 | ARK3 | BRI1 | receptor kinase 3 | -8.27 |
| MDP0000221530 | AT4G08850.1 |  | BRI1 | Leucine-rich repeat receptor-like protein kinase family protein | -4.63 |
| MDP0000298722 | AT4G08850.1 |  | BRI1 | Leucine-rich repeat receptor-like protein kinase family protein | -3.64 |
| MDP0000245676 | AT4G23180.1 | CRK10 | BRI1 | cysteine-rich RLK (RECEPTOR-like protein kinase) 10 | -3.70 |
| MDP0000280609 | AT1G70250.1 |  | BRI1 | receptor serine/threonine kinase, putative | -2.96 |
| MDP0000231625 | AT3G56100.1 | MRLK | BRI1 | meristematic receptor-like kinase | -1.74 |
| MDP0000637246 | AT3G05660.1 | RLP33 | BRI1 | receptor like protein 33 | -3.37 |
| MDP0000313836 | AT3G05660.1 | RLP33 | BRI1 | receptor like protein 33 | -3.94 |
| MDP0000127416 | AT3G51550.1 | FER | BRI1 | Malectin/receptor-like protein kinase family protein | -3.77 |
| MDP0000195256 | AT1G14390.1 |  | BRI1 | Leucine-rich repeat protein kinase family protein | -2.73 |
| MDP0000265003 | AT1G68400.1 |  | BRI1 | leucine-rich repeat transmembrane protein kinase family protein | -2.37 |
| MDP0000258524 | AT4G39400.1 | BRI1 | BRI1 | Leucine-rich receptor-like protein kinase family protein | -3.00 |
| MDP0000198878 | AT1G72300.1 |  | BRI1 | Leucine-rich receptor-like protein kinase family protein | -2.61 |
| MDP0000416388 | AT4G08850.1 |  | BRI1 | Leucine-rich repeat receptor-like protein kinase family protein | -2.62 |
| MDP0000950652 | AT1G72300.1 |  | BRI1 | Leucine-rich receptor-like protein kinase family protein | -1.74 |
| MDP0000248707 | AT5G01550.1 | LECRKA4.2 | BRI1 | lectin receptor kinase a4.1 | -1.90 |
| MDP0000922388 | AT3G56370.1 |  | BRI1 | Leucine-rich repeat protein kinase family protein | -1.81 |
| MDP0000181895 | AT1G45616.1 | RLP6 | BRI1 | receptor like protein 6 | -1.81 |
| MDP0000242000 | AT1G48480.1 | RKL1 | BRI1 | receptor-like kinase 1 | -1.48 |
| MDP0000276117 | AT1G66980.1 | SNC4 | BRI1 | suppressor of npr1-1 constitutive 4 | -1.45 |
| MDP0000190265 | AT2G01950.1 | BRL2 | BRI1 | BRI1-like 2 | -1.61 |
| MDP0000453826 | AT1G48480.1 | RKL1 | BRI1 | receptor-like kinase 1 | -1.72 |
| MDP0000241444 | AT2G26730.1 |  | BRI1 | Leucine-rich repeat protein kinase family protein | -1.42 |
| MDP0000203090 | AT1G79620.1 |  | BRI1 | Leucine-rich repeat protein kinase family protein | -1.63 |
| MDP0000259272 | AT3G24660.1 | TMKL1 | BRI1 | transmembrane kinase-like 1 | -1.41 |
| MDP0000277066 | AT4G03230.1 |  | BRI1 | S-locus lectin protein kinase family protein | -1.47 |
| MDP0000399163 | AT2G25470.1 | RLP21 | BRI1 | receptor like protein 21 | -1.45 |
| MDP0000172863 | AT1G27190.1 |  | BRI1 | Leucine-rich repeat protein kinase family protein | -1.16 |
| MDP0000297716 | AT1G72300.1 |  | BRI1 | Leucine-rich receptor-like protein kinase family protein | -1.71 |
| MDP0000200276 | AT4G21390.1 | B120 | BRI1 | S-locus lectin protein kinase family protein | -1.57 |
| MDP0000459059 | AT5G59010.1 |  | BSK | Protein kinase protein with tetratricopeptide repeat domain | 1.34 |
| MDP0000270591 | AT4G00710.1 | BSK3 | BSK | BR-signaling kinase 3 | 1.57 |
| MDP0000142512 | AT3G54030.1 |  | BSK | Protein kinase protein with tetratricopeptide repeat domain | -2.04 |
| MDP0000813397 | AT3G54030.1 |  | BSK | Protein kinase protein with tetratricopeptide repeat domain | -1.60 |
| MDP0000140641 | AT5G46570.1 | BSK2 | BSK | BR-signaling kinase 2 | -1.28 |
| MDP0000293071 | AT5G59010.1 |  | BSK | Protein kinase protein with tetratricopeptide repeat domain | -0.85 |
| MDP0000213245 | AT5G46570.1 | BSK2 | BSK | BR-signaling kinase 2 | -1.12 |
| MDP0000250138 | AT4G18710.1 | BIN2 | BIN2 | Protein kinase superfamily protein | 1.26 |
| MDP0000748952 | AT1G75080.1 | BZR1 | BZR1/2 | Brassinosteroid signalling positive regulator (BZR1) family protein | 1.81 |
| MDP0000410792 | AT1G75080.1 | BZR1 | BZR1/2 | Brassinosteroid signalling positive regulator (BZR1) family protein | 2.11 |
| MDP0000289260 | AT1G78700.1 | BEH4 | BZR1/2 | BES1/BZR1 homolog 4 | 1.14 |
