# Supplementary material for: Transcriptome analysis reveals the effects of sugar metabolism and auxin and cytokinin signaling pathways on root growth and development of grafted apple
Source: BMC Genomics. 2016 Feb 29;17:150. doi: 10.1186/s12864-016-2484-x (PMC4770530; doi:10.1186/s12864-016-2484-x)
Supplement: Additional file 5: — Selected root development-related genes from RNA sequencing data. (DOC 46 kb) [file 12864_2016_2484_MOESM5_ESM.doc]

**Additional file** **5 Selected differentially expressed genes related to cell division, differentiation and growth**

| **Apple genes Identification** | **Arabidopsis Homolog** | **Names** | **Annotation** | **log_2_(MB /WT)** |
| --- | --- | --- | --- | --- |
| MDP0000213592 | AT5G65460.1 | KAC2 | kinesin like protein for actin based chloroplast movement 2 | -0.86 |
| MDP0000286691 | AT2G13680.1 | CALS5 | callose synthase 5 | -1.32 |
| MDP0000216786 | AT2G26650.1 | AKT1 | K+ transporter 1 | -3.38 |
| MDP0000125070 | AT2G29570.1 | PCNA2 | Functionally interacts with POLH to repair DNA damaged by UVB damage | 1.05 |
| MDP0000313603 | AT2G29570.1 | PCNA2 | Functionally interacts with POLH to repair DNA damaged by UVB damage | 0.98 |
| MDP0000809276 | AT1G70210.1 | CYCD1;1 | CYCLIN D1;1 | -3.86 |
| MDP0000231873 | AT1G70210.1 | CYCD1;1 | CYCLIN D1;1 | -1.96 |
| MDP0000310564 | AT1G70210.1 | CYCD1;1 | CYCLIN D1;1 | -1.70 |
| MDP0000176105 | AT2G22490.1 | CYCD2;1 | Cyclin D2;1 | -2.43 |
| MDP0000286130 | AT4G34160.1 | CYCD3;1 | CYCLIN D3;1 | -2.17 |
| MDP0000155259 | AT5G67260.1 | CYCD3;2 | CYCLIN D3;2 | -0.97 |
| MDP0000286691 | AT2G13680.1 | CALS5 | callose synthase 5 | -1.32 |
| MDP0000568498 | AT2G47750.1 | GH3.9 | putative indole-3-acetic acid-amido synthetase GH3.9 | -2.26 |
| MDP0000786650 | AT2G46370.4 | FIN219 | Auxin-responsive GH3 family protein | -1.73 |
| MDP0000278275 | AT3G19820.1 | DWF1 | Encodes a gibberellin (GA) receptor ortholog of the rice GA receptor gene (OsGID1) | -0.72 |
| MDP0000682675 | AT3G19820.1 | DWF1 | Encodes a gibberellin (GA) receptor ortholog of the rice GA receptor gene (OsGID1) | 0.89 |
| MDP0000320017 | AT4G25810.1 | TCH4 | xyloglucan endotransglycosylase 6 | -0.12 |
| MDP0000225088 | AT5G57560.1 | TCH4 | xyloglucan endotransglycosylase 6 | -0.97 |
| MDP0000842877 | AT5G57560.1 | TCH4 | Xyloglucan endotransglucosylase/hydrolase family protein | -2.07 |
| MDP0000649866 | AT4G25810.1 | TCH4 | xyloglucan endotransglycosylase 6 | -1.75 |
