# Supplementary material for: Transcriptome analysis reveals the effects of sugar metabolism and auxin and cytokinin signaling pathways on root growth and development of grafted apple
Source: BMC Genomics. 2016 Feb 29;17:150. doi: 10.1186/s12864-016-2484-x (PMC4770530; doi:10.1186/s12864-016-2484-x)
Supplement: Additional file 6: — Selected differentially expressed genes related to cell division, differentiation and growth. (DOC 39 kb) [file 12864_2016_2484_MOESM6_ESM.doc]

**Additional file 6 Selected root development-related genes from RNA sequencing data**

| **Apple genes Identification** | **Arabidopsis Homolog** | **Names** | **Annotation** | **log_2_(MB/WT)** |
| --- | --- | --- | --- | --- |
| MDP0000242242 | AT2G01830.1 | WOL1 | CHASE domain containing histidine kinase protein | 1.46 |
| MDP0000258078 | AT5G35750.1 | AHK2 | histidine kinase 2 | 0.52 |
| MDP0000181429 | AT1G27320.1 | AHK3 | histidine kinase 3 | -9.72 |
| MDP0000172418 | AT3G12280.2 | RBR1 | retinoblastoma-related 1 | 1.22 |
| MDP0000258414 | AT1G49620.1 | KRP7 | Cyclin-dependent kinase inhibitor family protein | 0.94 |
| MDP0000256052 | AT2G35350.1 | PLL1 | poltergeist like 1 | -1.53 |
| MDP0000142574 | AT2G19580.1 | TET2 | tetraspanin2 | -1.07 |
| MDP0000125975 | AT3G62980.1 | TIR1 | F-box/RNI-like superfamily protein | 1.38 |
| MDP0000498419 | AT3G62980.1 | TIR1 | F-box/RNI-like superfamily protein | 2.69 |
| MDP0000324919 | AT1G04240.1 | SHY2 | AUX/IAA transcriptional regulator family protein | 1.55 |
| MDP0000945260 | AT1G04240.1 | SHY2 | AUX/IAA transcriptional regulator family protein | 0.56 |
| MDP0000208345 | AT1G04240.1 | SHY2 | AUX/IAA transcriptional regulator family protein | 0.21 |
| MDP0000295589 | AT1G04240.1 | SHY2 | AUX/IAA transcriptional regulator family protein | 0.56 |
| MDP0000303142 | AT1G04240.1 | SHY2 | AUX/IAA transcriptional regulator family protein | 0.28 |
| MDP0000885425 | AT2G21050.1 | LAX2 | like AUXIN RESISTANT 2 | -2.24 |
| MDP0000155113 | AT2G38120.1 | AUX1 | Transmembrane amino acid transporter family protein | -0.89 |
| MDP0000749280 | AT2G38120.1 | AUX1 | Transmembrane amino acid transporter family protein | -0.93 |
| MDP0000080407 | AT2G38120.1 | AUX1 | Transmembrane amino acid transporter family protein | 1.54 |
| MDP0000175425 | AT2G38120.1 | AUX1 | Transmembrane amino acid transporter family protein | 1.40 |
| MDP0000167283 | AT5G11030.3 | ALF4 | aberrant lateral root formation 4 | 0.72 |
| MDP0000130583 | AT3G16500.1 | PAP1 | phytochrome-associated protein 1 | -0.81 |
| MDP0000753736 | AT3G16500.1 | PAP1 | phytochrome-associated protein 1 | -1.74 |
| MDP0000164095 | AT3G16500.1 | PAP1 | phytochrome-associated protein 1 | -0.97 |
| MDP0000155098 | AT1G62440.1 | LRX2 | leucine-rich repeat/extensin 2 | -3.65 |
| MDP0000895663 | AT5G05730.1 | TRP5 | anthranilate synthase alpha subunit 1 | -1.88 |
| MDP0000256052 | AT2G35350.1 | PLL1 | poltergeist like 1 | -1.35 |
| MDP0000142574 | AT2G19580.1 | TET2 | tetraspanin2 | -1.02 |
| MDP0000165587 | AT4G37650.1 | SHR | GRAS family transcription factor | -1.20 |
| MDP0000124971 | AT5G53970.1 | ALF1 | Tyrosine transaminase family protein | -2.38 |
| MDP0000167283 | AT5G11030.3 | ALF4 | aberrant lateral root formation 4 | 0.72 |
