# Supplementary material for: Transcriptome analysis reveals the effects of sugar metabolism and auxin and cytokinin signaling pathways on root growth and development of grafted apple
Source: BMC Genomics. 2016 Feb 29;17:150. doi: 10.1186/s12864-016-2484-x (PMC4770530; doi:10.1186/s12864-016-2484-x)
Supplement: Additional file 7: — Selected differentially expressed genes related to salicylic and jasmonic acid signaling and nitrogen metabolism. (DOC 48 kb) [file 12864_2016_2484_MOESM7_ESM.doc]

**Additional file 7 Selected differentially expressed genes related to salicylic and jasmonic acid signaling and nitrogen metabolism**

| **Apple Genes Identification** | **Arabidopsis Homolog** | **Names** | **Annotation** | **log_2_(MB/WT)** |
| --- | --- | --- | --- | --- |
| **Salicylic acid signaling** | |  |  |  |
| MDP0000167136 | AT2G41370.1 | BOP2 | Ankyrin repeat family protein / BTB/POZ domain-containing protein | -1.13 |
| MDP0000597773 | AT1G58330.1 | ZW2 | transcription factor-related | 1.86 |
| MDP0000261154 | AT4G37730.1 | bZIP7 | basic leucine-zipper 7 | 1.27 |
| MDP0000298108 | AT1G10560.1 | PUB18 | plant U-box 18 | 1.27 |
| MDP0000277999 | AT1G77920.1 |  | bZIP transcription factor family protein | -0.91 |
| MDP0000295390 | AT4G25780.1 |  | CAP (Cysteine-rich secretory proteins, Antigen 5, and Pathogenesis-related 1 protein) superfamily protein | -4.33 |
| MDP0000711379 | AT4G33720.1 |  | CAP (Cysteine-rich secretory proteins, Antigen 5, and Pathogenesis-related 1 protein) superfamily protein | -4.22 |
| **Jasmonic acid signaling** | |  |  |  |
| MDP0000277815 | AT2G39940.1 | COI1 | RNI-like superfamily protein | 0.96 |
| MDP0000802641 | AT3G48520.1 | CYP94B3 | cytochrome P450, family 94, subfamily B, polypeptide 3 | -4.97 |
| MDP0000786650 | AT2G46370.4 | FIN219 | Auxin-responsive GH3 family protein | -1.73 |
| **Nitrogen metabolism** | |  |  |  |
| MDP0000209368 | AT4G37560.1 |  | Acetamidase/Formamidase family protein | -2.14 |
| MDP0000255705 | AT4G37560.1 |  | Acetamidase/Formamidase family protein | -1.66 |
| MDP0000268029 | AT1G26340.1 | CB5-A | cytochrome B5 isoform A | 0.98 |
| MDP0000165379 | AT3G01500.2 | SABP3 | carbonic anhydrase 1 | -10.12 |
| MDP0000215729 | AT3G01500.2 | SABP3 | carbonic anhydrase 1 | -10.30 |
| MDP0000194249 | AT5G14740.1 | CA2 | carbonic anhydrase 2 | 7.57 |
| MDP0000177116 | AT5G14740.1 | CA2 | carbonic anhydrase 2 | -2.34 |
| MDP0000166668 | AT5G14740.1 | CA2 | carbonic anhydrase 2 | -1.52 |
| MDP0000119793 | AT1G70410.1 | CA4 | beta carbonic anhydrase 4 | -1.70 |
| MDP0000128727 | AT3G23490.1 | CYN | cyanase | -1.31 |
| MDP0000523096 | AT3G27010.1 | TCP20 | TEOSINTE BRANCHED 1, cycloidea, PCF (TCP)-domain family protein 20 | 1.10 |
| MDP0000688645 | AT4G27450.1 |  | Aluminium induced protein with YGL and LRDR motifs | 1.35 |
| MDP0000744728 | AT3G47340.1 | ASN1 | glutamine-dependent asparagine synthase 1 | -2.15 |
| MDP0000119630 | AT3G47340.1 | ASN1 | glutamine-dependent asparagine synthase 1 | -2.37 |
| MDP0000096208 | AT3G47340.1 | ASN1 | glutamine-dependent asparagine synthase 1 | -1.56 |
| MDP0000213005 | AT5G07440.1 | GDH2 | glutamate dehydrogenase 2 | -1.32 |
| MDP0000499580 | AT5G07440.1 | GDH2 | glutamate dehydrogenase 2 | -0.96 |
| MDP0000499579 | AT5G07440.1 | GDH2 | glutamate dehydrogenase 2 | -0.87 |
| MDP0000945325 | AT5G07440.1 | GDH2 | glutamate dehydrogenase 2 | -9.87 |
| MDP0000293627 | AT1G11860.1 |  | Glycine cleavage T-protein family | -1.05 |
