# Supplementary material for: Transcriptome analysis reveals the effects of sugar metabolism and auxin and cytokinin signaling pathways on root growth and development of grafted apple
Source: BMC Genomics. 2016 Feb 29;17:150. doi: 10.1186/s12864-016-2484-x (PMC4770530; doi:10.1186/s12864-016-2484-x)
Supplement: Additional file 8: — Gene-specific primers used for quantitative real-time PCR. (DOC 37 kb) [file 12864_2016_2484_MOESM8_ESM.doc]

**Additional file 8 Gene-specific primers used for quantitative real-time PCR**

| **Apple Genes Identification** | **Arabidopsis Homolog** | **Primers (5'-3')** |
| --- | --- | --- |
| MDP0000759646 | SDH | F:AATCCTGCAATGGCGTGGTTAGAG  R:AATGCCGACAGCCTTAATCCGAATT |
| MDP0000250070 | SUS4 | F:CTCAAGCGTGTTAAGCAACAG  R:CTGAATGGAACACGAAGAATATC |
| MDP0000293468 | PCK1 | F:ACGCAGAAGAAGGACAATGGAGTG  R:TCAGAGAGGGTGACGGCAAAGG |
| MDP0000931334 | ACLA-3 | F:AGACGTAGCTGCTACATTCAGTGGA  R:AATTCCGGTCATTGTTGCCTCAGG |
| MDP0000138035 | PIN1 | F: GCGGGATCCATTGTCTCCATCC  R: CTGAGACCGCCGCGAGAAAATA |
| MDP0000809218 | AFB5 | F:TCTGCAAGTCCTGGTACCGC  R: GCTTGCCCTTGATGGTCACG |
| MDP0000456476 | IAA9 | F: TGAGGAGGAGGGTCAGAGCG  R: TCAGAGACAGCAGAGACCTTCGA |
| MDP0000543718 | IAA29 | F: ACGAAAACGATGGCTCAGCA  R: GCAGCACCACCCTCTTTGCT |
| MDP0000267175 | ABCG14 | F: GTCATTCTGCGGGGCCATCA  R: TGCTGCACTTTCTCGTCCCG |
| MDP0000123837 | AHP2 | F: AGCTCTGCACTGAAGAACAAGC  R: TCGTAGGAATTGACCCGCCA |
| MDP0000250737 | ARR3 | F: CGCCGGTCCAAATTTGCCAA  R: CTTTCTGCGGGCGGTGTTTT |
| MDP0000842877 | TCH4 | F: TGGAACGCTGATGACTGGGC  R: CCGAAGACCACCAAATGCAAGC |
| MDP0000165587 | SHR | F: ACTGATGAGACGCCGCACTT  R: CCATCCTCGCGCCAATTTCC |
| MDP0000324919 | SHY2 | F: AAATGGAAGGCAAGGCACATGA  R: TTCATCTCTCCCTGGCAACCC |
| MDP0000172418 | RBR | F: TCACTGCCATCAACAACCGCT  R: GGCCACCATCGAATCGCTGA |
| MDP0000167283 | ALF4 | F: TTGCGCCTCTTCTAACTGGGC  R: CAACTCCAAAGACCGGCCCTT |
